# STAT2 Signaling Regulates Macrophage Phenotype During Influenza and Bacterial Super-Infection

**DOI:** 10.3389/fimmu.2018.02151

**Published:** 2018-09-25

**Authors:** Radha Gopal, Benjamin Lee, Kevin J. McHugh, Helen E. Rich, Krishnaveni Ramanan, Sivanarayana Mandalapu, Michelle E. Clay, Philip J. Seger, Richard I. Enelow, Michelle L. Manni, Keven M. Robinson, Javier Rangel-Moreno, John F. Alcorn

**Affiliations:** ^1^Department of Pediatrics, Children's Hospital of Pittsburgh, University of Pittsburgh Medical Center, Pittsburgh, PA, United States; ^2^Department of Pediatrics, University of Vermont College of Medicine, Burlington, VT, United States; ^3^Department of Medicine, Dartmouth Medical School, Lebanon, PA, United States; ^4^Department of Medicine, University of Pittsburgh Medical Center, Pittsburgh, PA, United States; ^5^Division of Allergy, Immunology and Rheumatology, Department of Medicine, University of Rochester Medical Center, Rochester, NY, United States

**Keywords:** influenza, Staphylococcus aureus, super-infection, STAT2, macrophages, lung, pneumonia

## Abstract

Influenza is a common respiratory virus that infects between 5 and 20% of the US population and results in 30,000 deaths annually. A primary cause of influenza-associated death is secondary bacterial pneumonia. We have previously shown that influenza induces type I interferon (IFN)-mediated inhibition of Type 17 immune responses, resulting in exacerbation of bacterial burden during influenza and *Staphylococcus aureus* super-infection. In this study, we investigated the role of STAT2 signaling during influenza and influenza-bacterial super-infection in mice. Influenza-infected *STAT2*^−/−^ mice had increased morbidity, viral burden, and inflammation when compared to wild-type mice. Despite an exaggerated inflammatory response to influenza infection, we found increased bacterial control and survival in STAT2 deficient mice during influenza-MRSA super-infection compared to controls. Further, we found that increased bacterial clearance during influenza-MRSA super-infection is not due to rescue of Type 17 immunity. Absence of STAT2 was associated with increased accumulation of M1, M2 and M1/M2 co-expressing macrophages during influenza-bacterial super-infection. Neutralization of IFNγ (M1) and/or Arginase 1 (M2) impaired bacterial clearance in *Stat2*^−/−^ mice during super-infection, demonstrating that pulmonary macrophages expressing a mixed M1/M2 phenotype promote bacterial control during influenza-bacterial super-infection. Together, these results suggest that the STAT2 signaling is involved in suppressing macrophage activation and bacterial control during influenza-bacterial super-infection. Further, these studies reveal novel mechanistic insight into the roles of macrophage subpopulations in pulmonary host defense.

## Introduction

Influenza A virus infection causes significant mortality and morbidity worldwide. It is estimated that influenza infection causes 3 million hospitalizations and 250,000 deaths, globally each year (1). The innate immune system senses invading influenza viruses through a variety of pattern recognition receptors, inducing the production of inflammatory cytokines and chemokines, including type I (IFNα/β) and type III interferons (IFNλ) (2–4). Type I IFNs (IFNα/β) bind to the heterodimer of IFNaR1 and IFNaR2 receptors, associated with Jak1 and Tyk2 kinases, respectively, to produce signaling effects during influenza infection (5). Signaling through IFNaR1/2 leads to phosphorylation of STAT1 and STAT2, activating downstream complex formation with IRF9, and transcription of IFN stimulated genes (ISGs), thereby controlling viral burden. Type II IFN (IFNγ) activates only STAT1 signaling through IFNγR1 and IFNγR2 and plays a role in cell-mediated immunity (6, 7). Type III IFNs signal through IFNLR and IL10R2 to induce ISGs similar to those induced by type I IFN (7). Type I, type II and type III IFNs have been shown to have important roles during influenza infection (7–9). In the absence of type I or type II IFN during influenza infection, increased granulocytic or lymphocytic inflammation, respectively, has been reported (7). Absence of STAT1 signaling specifically resulted in an increased granulocytic and Th2-skewed response (7), and both STAT1 and STAT2 were crucial for viral control and survival in mice (9).

Secondary bacterial pneumonia during influenza infection is a common cause of influenza-associated hospitalization and mortality during both seasonal and pandemic outbreaks (10, 11). During the 1918 influenza pandemic, *Streptococcus pneumoniae* was the most common bacteria isolated from influenza-bacteria super-infected patients (10). However, recent reports have shown that *Staphylococcus aureus* is now the most frequent super-infecting bacteria (10, 12). We have shown that during influenza-bacterial super-infection, influenza-induced type I IFN inhibited *S. aureus*-induced Type 17 immunity and associated antimicrobial peptide (AMP) production (13, 14). Further, we and others have shown that mice lacking the type I IFN receptor cleared *S. aureus* and *S. pneumoniae* better than wild-type (WT) mice during super-infection (13, 15). While influenza infection alters host defense to *S. aureus*, the converse is also likely true; *S. aureus* increases influenza burden in the lung, possibly by affecting STAT1-STAT2 dimerization during super-infection (13, 16). These data suggest that influenza-induced STAT1 and STAT2 signaling is critical to mediating susceptibility to secondary bacterial pneumonia. We have recently shown that STAT1 is involved in increasing bacterial burden through suppression of the Type 17 immune response during influenza-bacterial super-infection (17). However, little is known regarding the specific role of STAT2 in super-infection.

Since type I and type III IFN signaling relies on STAT2, while type II IFN signals solely through STAT1, examination of STAT2 deficiency enables a more targeted evaluation of type I and type III IFN-mediated immune responses. In this study, we investigated the role of STAT2 signaling during influenza infection and influenza-bacterial super-infection by infecting WT and *Stat2*^−/−^ mice with influenza A/PR/8/34 followed by MRSA (USA300) challenge, and evaluating subsequent survival, morbidity, viral and bacterial burden, and inflammatory responses. We then elucidated differential host responses in *Stat2*^−/−^ mice using RNA expression, flow cytometry, immunohistochemistry and *in vitro* macrophage culture. Further, we investigated the role of hematopoietic and non-hematopoietic STAT2 signaling during influenza-bacterial super-infection. These studies are the first to define the role of STAT2 signaling in influenza, bacterial super-infection and identify a novel macrophage-dependent mechanism of susceptibility to secondary bacterial pneumonia.

## Materials and methods

### Mice

WT C57BL/6 (6 to 8-week-old) mice were purchased from Taconic Farms (Germantown, NY). *Stat2*^−/−^ mice on C57BL/6 background were a kind gift from Dr. Christian Schindler, Columbia University, NY (18), and colonies were subsequently maintained under specific pathogen-free conditions. *In vivo* studies were performed on age matched adult male mice, unless otherwise indicated. All experiments were approved by the University of Pittsburgh IACUC (19).

### Murine infections

Influenza A/PR/8/34 (influenza H1N1) was propagated in chicken eggs as described (20). Mice were infected with 100 plaque-forming units (PFU) of influenza in 40 μl of sterile PBS, unless otherwise noted. MRSA, USA 300, was provided by Dr. Alice Prince, Columbia University, NY. MRSA stocks were grown overnight in casein hydrolysate yeast extract-containing modified broth medium at 37°C and diluted to an inoculum of 5 × 10^7^ CFU in 50 μl of sterile PBS. MRSA dosing was calculated using OD_660_ measurement of overnight cultures and application of an extinction coefficient. For survival experiments, 2 × 10^8^ CFU of MRSA were delivered. All infections were performed on isoflurane-anesthetized mice via oropharyngeal aspiration. For super-infection experiments, mice were challenged with influenza or vehicle and then infected with MRSA or vehicle on day 6 after influenza infection (13, 21, 22). Mouse tissues were collected 24 h after MRSA or vehicle challenge. To neutralize IFNγ, mice were treated with 300 μg anti-IFNγ (XMG1.2) antibody in 200 μl sterile PBS (BioXCell, West Lebanon, NH) or rat IgG isotype control via intraperitoneal (IP) injection on days 4 and 6 post-influenza infection. To neutralize arginase, mice were treated with 100 μg N-^−^hydroxy-nor-L-arginine (nor-NOHA) via IP injection on days −1, 0, 3, and 6 post-influenza.

### Measurement of lung inflammation

Lungs were perfused with 1 ml of sterile PBS and cell differential counts were performed on cytospin smears from bronchoalveolar lavage (BAL) fluid stained with Protocol Hema 3 staining (Fisher Scientific, Kalamazoo, MI). The cranial lobe of the right lung was homogenized in sterile PBS by mechanical grinding, for quantification of bacterial burden by plating serial dilutions and for cytokine production measurement by Lincoplex (Millipore, Billerica, MA), Bio-plex (Bio-Rad, Hercules, CA), or ELISA (R&D Systems, Minneapolis, MN). The middle and caudal lobes of the right lung were snap-frozen and homogenized in liquid nitrogen for RNA isolation using the Absolutely RNA Miniprep Kit (Agilent Technologies, Santa Clara, CA). Gene expression was analyzed by RT-PCR utilizing commercially available Taqman primer and probe sets (Applied Biosystems, Foster City, CA). Fold changes in mRNA expression were calculated using the ΔΔCT method, and were normalized to the endogenous housekeeping gene hypoxanthine-guanine phosphoribosyltransferase (HPRT). The left lobe of the lung was pressure inflated and fixed in 10% neutral-buffered formalin for histology, or collected in DMEM media for flow cytometry. Histology was scored by a sample blinded pathologist.

### Flow cytometry

Mouse lungs were aseptically dissected into small sections, digested for 30 min at 37°C in 1 mg/mL collagenase media, and passed through 70 μm filters (23). Single cell suspensions were stained with anti-CD3 (145-2C11), CD4 (RM4-5), CD11b (M1/70), Ly6C (HK1.4), CD80(16-10A1), and macrophage galactose lectin (MGL) (LOM-14). After the singlets were gated from total cells, macrophages were gated for CD11b^+^Ly6C^+^ cells. Further, these cells were gated for CD80 and MGL to determine macrophage phenotype as M1 (CD80^+^) and/or M2 (MGL^+^). The frequency of CD11b^+^Ly6C^+^, CD11b^+^Ly6C^+^ CD80^+^, CD11b^+^ Ly6C^+^ CD80^+^ MGL^+^, and CD11b^+^ Ly6C^+^ MGL^+^ cells were calculated from the frequency of total cells. For intracellular staining, cells were stimulated for 4 h with 50 ng/mL of phorbol myristate acetate (PMA) and 750 ng/mL ionomycin (Sigma-Aldrich, St Louis, MO) with Golgi plug (BD Pharmingen, San Diego, CA) added 1 h into stimulation. After stimulation, cells were surface stained, permeabilized with cytofix-cytoperm solution (BD Pharmingen, San Diego, CA), and stained with antibodies specific for IL-17 (TC11-18H10), IL-22 (Poly5164) for 30 min at 4°C. The percentage of IL-17^+^ and IL-22^+^ cells were determined from gating on CD3^+^CD4^+^ T cells. Cells were collected in a Becton Dickinson FACS Aria flow cytometer with FACS Diva software (BD, Franklin Lakes, NJ). Flow cytometric analysis was performed using FlowJo (Tree Star, Ashland, OR).

### Immunohistochemistry

Left lung lobes were perfused and stored in 10% neutral buffered formalin. Paraffin-embedded sections were stained with hematoxylin and eosin (H&E) and pulmonary inflammation was evaluated by microscopy. For immunofluorescent staining, formalin-fixed lung sections were incubated at 60°C, and quickly immersed in xylene to remove paraffin. Sections were then hydrated in 96% alcohol and PBS. Antigen retrieval was performed by incubating slides in boiling Dako Target Retrieval Solution (S1699, DAKO Cytomation), followed by blocking with 5% (v/v) normal donkey (017-000-121, Jackson ImmunoResearch Laboratories) serum and Fc block (BD Pharmingen, San Diego, CA). Sections were stained with antibodies against inducible NO synthase (iNOS) (goat anti-mouse iNOS, clone M-19; Santa Cruz Biotechnology Inc.), F4/80 (clone Cl:A3-1, MCA497GA, Bio-Rad), and arginase-1 (Arg1) (rabbit anti–arginase I, clone H-52; Santa Cruz Biotechnology Inc.). Primary antibodies were detected with Alexa Fluor 568-donkey anti-goat Ig G (H+L) cross adsorbed (A-11057; Invitrogen) to detect iNOS, donkey anti-rabbit Ig G (H+L) antibody conjugated to FITC (711-095-152, Jackson Immuno Research Laboratories) to visualize Arg1. Slides were incubated with biotin-F(ab')_2_ donkey anti-rat (712-006-153 Jackson Immuno Research Laboratories), followed by Cy5-Streptavidin (405209, Biolegend) to reveal the location of F4/80^+^ macrophages. Vectashield anti-fade mounting medium with DAPI (H-1200, Vector Laboratories) was used to counterstain tissues and to detect nuclei. Images were obtained with a Zeiss Axioplan 2 microscope and recorded with a Zeiss AxioCam digital camera. iNOS, F4/80, and Arg1 positive cells were enumerated in three random 200x fields per lung sample, and the average number of iNOS^+^, Arg1^+^ or double positive macrophages was calculated. Samples were analyzed in a blinded fashion.

### Arginase-1 activity determination

Arginase-1 activity was quantified in lung BAL using the QuantiChrom Arginase Assay Kit as per the manufacturer's instructions (BioAssay Systems). In BAL samples, total protein was measured by using BCA Protein Assay kit (Pierce Biotechnology, Rockford, IL). Protein concentration was adjusted to 100 μg/ml per sample. Arg-1 activity is expressed as U/l of sample.

### Nitrite quantitation

Nitrite was quantified in BAL samples by using Griess reagent as per the manufacturer's instructions (Promega Corporation).

### RNAseq analysis

RNA was isolated from C57BL/6 and Stat2^−/−^ mouse lungs using the Agilent RNA miniprep kit. RNA integrity was determined using an Agilent 2100 bio analyzer. mRNA was purified by using Sera-Mag Oligo(dT) Beads, fragmented with magnesium-catalyzed hydrolysis, and reverse transcribed into cDNA using random primers (Superscript II; Invitrogen). Then, cDNA underwent end repair with T4 DNA polymerase and Klenow DNA polymerase, followed by the addition of “A” bases to the 3′ end and ligation to adaptor oligonucleotides. Products from the ligation were run on a 2% agarose gel. A gel slice consisting of the 200 bp region (±25 bp) was excised and used as a template for PCR amplification. The final PCR product was purified, denatured with 2 N NaOH, and diluted to 10–12 pM prior to cluster amplification on a single-read flow cell v4, as outlined in the Single-Read Cluster Generation Kit v4 (Illumina). The flow cell was sequenced on an Illumina Genome Analyzer II. The data were analyzed as previously described (24). Full sequencing data has been uploaded to the National Center for Biotechnology Information Gene Expression Omnibus, GSE119029.

### Bone marrow-derived macrophage (BMDM) and dendritic cell (BMDC) generation

Bone marrow cells were isolated from the femurs and tibias of mice, and grown for 7 days in complete DMEM media supplemented with 20 ng/mL GM-CSF (PeproTech, NJ), as previously described (25). On day 7, non-adherent cells (BMDCs) were collected, and adherent cells (BMDMs) were recovered using cell scraper (Genemate, Kaysville, UT) or by gentle mechanical scraping.

### Bone marrow-derived macrophage (BMDM) and dendritic cell (BMDC) stimulation

Bone marrow derived cells were plated to 1 × 10^6^ cells/mL and cells in 1 mL of media in 24 well tissue culture-treated plates, rested overnight at 37°C in 5% CO_2_, then treated with IFN-β (10 units/mL) or IFN-γ (10 ng/mL) (R&D Systems, MN). Twenty-four hours later, cell culture supernatants were harvested and cells were lysed in RLT buffer and frozen at −80°C for RNA extraction using the RNAeasy mini kit (QIAGEN, Germantown, MD. Gene expression was analyzed as described above.

### BMDM phagocytosis and killing assay

BMDMs were incubated with fluorescein isothiocyanate or Alexa Fluor-647 (Molecular Probes) —labeled MRSA (10 MOI) for 30 min. After washing, flow cytometry was performed to determine bacterial uptake. To determine bacterial killing by macrophages, BMDMs were incubated with MRSA for 1 h. Cells were washed with PBS and the remaining macrophages were incubated with gentamycin (300 μl/ml) for 15 min to kill extracellular bacteria. Cells were washed and incubated for an additional hour to determine killing of intracellular bacteria. Cells were washed and lysed with 0.5 ml of 0.02% Triton X-100 in PBS, and plated to determine the percentage of intracellular bacterial killing.

### Bone marrow chimera (BMC) mice

To generate BMC mice, C57BL/6 (Thy1.1), C57BL/6 (Thy1.2), and *Stat2*^−/−^ (Thy 1.2) mice were fed with Sulfa-Trimm diet containing 1.2% sulfamethoxazole and 0.2% trimethoprim for 2 weeks before irradiation (26, 27). Mice were sub-lethally irradiated twice with two doses of 600 rad delivered 4 h apart. Mice were reconstituted with 1 × 10^7^ bone marrow cells from either C57BL/6 (Thy1.1), C57BL/6 (Thy1.2), or *Stat2*^−/−^ (Thy 1.2) mice as previously described (26). Mice were allowed to reconstitute for 9 weeks.

### Statistical analysis

Data were analyzed using GraphPad Prism software. Experiments were repeated 2 to 5 times. All data are presented as mean ± SEM, unless otherwise noted. Significance was determined by unpaired Student's *t* test or one-way ANOVA followed by *post-hoc* Bonferroni or Tukey's test for multiple comparisons. Mortality data was analyzed by Log-rank (Mantel-Cox) test.

## Results

### Influenza severity is increased in *stat2^−/−^* mice compared to WT

**Figure 1:**
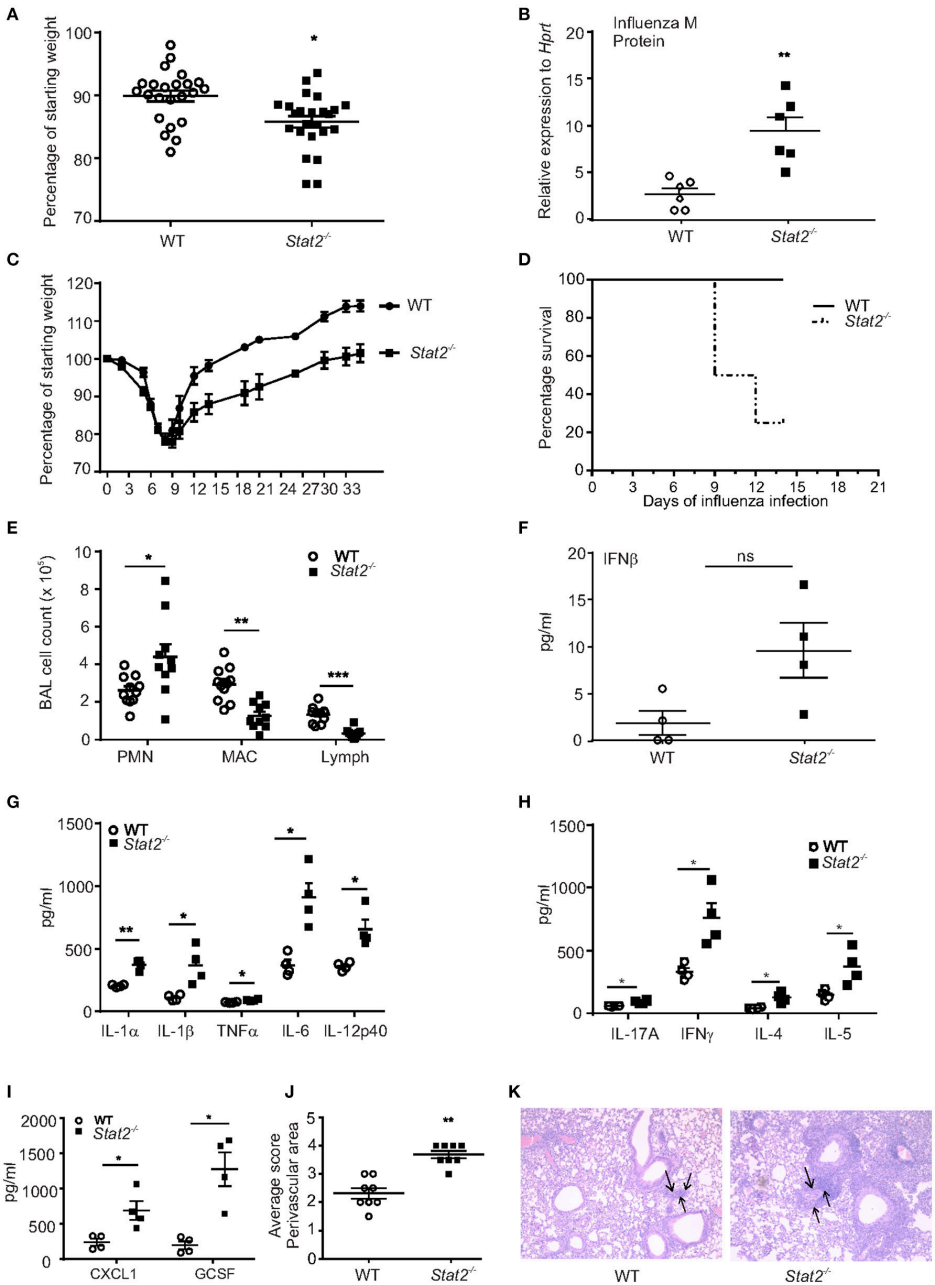
*Stat2*^−/−^ mice have increased susceptibility to influenza infection. **(A)** WT, *Stat2*^−/−^ male 6–8 weeks mice were infected with 100 PFU of influenza, percentage weight loss on day 6 of influenza infection, *N* = 22–24 per group. **(B)** Viral burden was measured by influenza M protein expression 6 days following influenza infection in whole lung, *N* = 6 per group. **(C)** Weight loss during 35 days following influenza infection, *N* = 8 per group. **(D)** Percentage survival following influenza infection in females, *N* = 8 per group. **(E)** BAL samples collected on day 6 following influenza infection from mice lungs, cytospin the cells and differential counts were made *N* = 10–12 per group. Right upper lung lobes were homogenized in PBS, **(F)** IFNβ levels were measured in BAL samples by using ELISA, *N* = 4 per group. **(G)** IL-1α, IL-1β, TNFα, IL-6, and IL-12p40, **(H)** IL-17A, IFNγ, IL-4, and IL-5 **(I)** CXCL1, and GCSF levels were measured by Luminex assay, *N* = 4 per group. Representative data shown from 3 or more experiments are shown **(J)** On day 6 post infection, lungs were fixed in 10% formalin, embedded in paraffin, stained with H&E, lung perivascular areas (arrows) were scored. **(K)** Representative histology pictures are shown. Original magnification for H&E sections × 100. Data are represented as mean±SEM, two tailed Student's *t*-test, ^*^p < 0.05, ^**^p < 0.01, ^***^p < 0.001, ns, not significant.

To determine the role of STAT2 signaling during influenza infection, WT and *Stat2*^−/−^ mice were infected with influenza. By day 6 of influenza infection, influenza-challenged *Stat2*^−/−^ mice showed significantly more weight loss than WT mice and increased lung viral burden (Figures 1A,B). In males, infection was sub-lethal in both *Stat2*^−/−^ and WT mice, but *Stat2*^−/−^ mice showed markedly delayed recovery, requiring 35 days to return to baseline weight, versus 14 days for WT mice (Figure 1C). In females, *Stat2*^−/−^ mice showed increased mortality compared to WT during influenza infection (Figure 1D). Next, we determined the cellular inflammatory response to influenza infection in WT and *Stat2*^−/−^ mice. There was a significantly greater number of polymorphonuclear cells (PMN, neutrophils) observed in bronchoalveolar lavage (BAL) from *Stat2*^−/−^ mice when compared to WT (Figure 1E). Interestingly, *Stat2*^−/−^ mice had significantly fewer numbers of macrophages and lymphocytes in BAL compared to WT mice (Figure 1E).

Next, we determined whether type I IFN levels were altered during influenza infection. We found a trend toward increased levels of IFNβ in *Stat2*^−/−^ mice when compared to WT mice during primary influenza infection (Figure 1F). Influenza infection induced a variety of inflammatory cytokines such as IL-1β, IL-6, TNFα, IL-8, CCL2, CCL5, and CXCL10 from epithelial and immune cells (2). We found an increase in the levels of the inflammatory cytokines IL-1α, IL-1β, TNFα, IL-6, IL-12p40, IL-17, and IFNγ, and increased levels of the Type 2 cytokines IL-4 and IL-5, in influenza-infected *Stat2*^−/−^ mice when compared to WT mice (Figures 1G,H).

It has been shown that IL-17 signaling induces the chemokines CXCL1, CXCL2, CXCL5, and CXCL8 to mediate granulopoiesis and increased neutrophil recruitment to mucosal sites (28, 29). Consistent with the increase in PMNs, we observed an increase in CXCL1 and the granulopoietic factor, G-CSF in *Stat2*^−/−^ when compared to WT mice (Figure 1I). Further, scoring of pulmonary inflammation indicated an increase in perivascular infiltration of cells in the lungs of *Stat2*^−/−^ mice when compared to WT mice (Figures 1J,K). Together, these data demonstrate that influenza-infected *Stat2*^−/−^ mice have more severe influenza infection, which is associated with an increased inflammatory response, compared to WT mice.

### *Stat2^−/−^* mice are rescued from impaired bacterial clearance during influenza/MRSA super-infection

**Figure 2:**
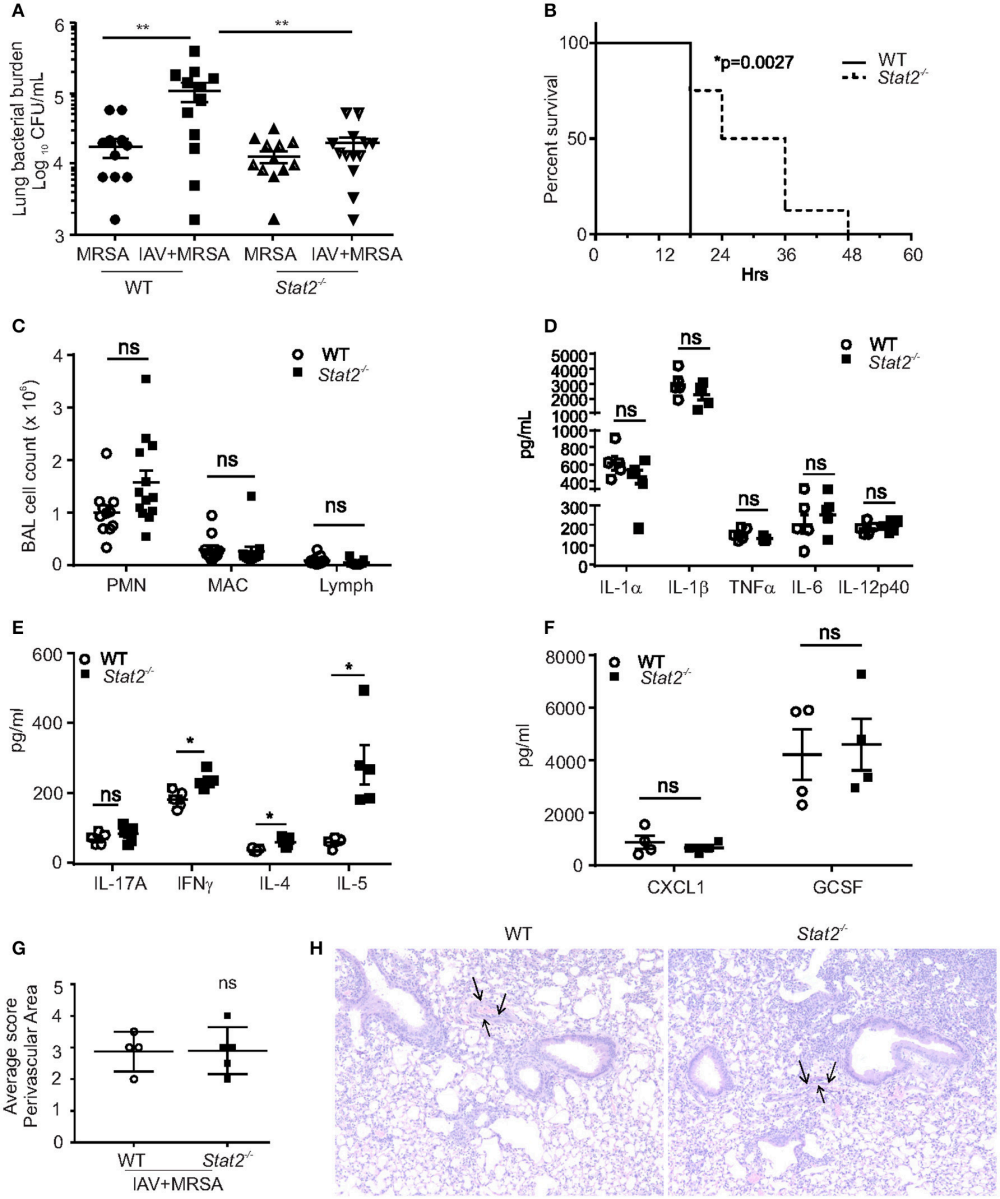
*Stat2*^−/−^ are rescued from impaired bacterial clearance from the lung following influenza infection. **(A)** WT or *Stat2*^−/−^ mice were infected with 100 PFU of influenza for 6 days then challenged with 5 × 10^7^ cfu of MRSA for one additional day. Right upper lung lobes were homogenized in PBS and bacterial burden was determined. *N* = 12–13 per group. **(B)** Adult female mice were infected with 66 PFU influenza A PR/8/H1N1 followed by challenge with 2 × 10^8^ CFU MRSA and the survival was determined, *N* = 7–8 per group. **(C)** BAL samples collected from co-infected B6 and *Stat2*^−/−^ mice, cytospin the cells and differential counts were made, *N* = 11–13 per group. Right Upper lung lobes were homogenized in PBS, and **(D)** IL-1α, IL-1β, TNFα, IL-6, IL-12p40, **(E)** IL-17A, IFNγ, IL-4, IL-5 **(F)** CXCL1, and GCSF levels were measured by luminex assay, *N* = 4 per group. Representative data shown from three or more experiments. Lungs were fixed in 10% formalin, embedded in paraffin, **(G)** perivascular areas (arrows) were scored in formalin fixed lungs by staining with H&E **(H)** representative figures are shown, *N* = 4 per group. Original magnification for H&E sections × 100. Data are represented as mean±SEM. Data analyzed using 2-tailed Student's *t*-test or One way ANOVA followed by Bonferroni test for multiple comparisons, ^*^p < 0.05, ^**^p < 0.01, ns, not significant.

We have previously shown that influenza-associated IFNβ attenuated host bacterial defense due to suppression of Type 17 immunity (13, 14). Also, we recently identified that Type 17 immunity is rescued in the absence of STAT1 signaling during influenza-bacterial super-infection (17). In this study, we examined the effects of MRSA challenge on day 6 of influenza infection in WT and *Stat2*^−^^/−^ mice. One day following MRSA challenge, super-infected WT mice had significantly increased lung bacterial burden compared to mice infected with MRSA alone, demonstrating impaired bacterial clearance from the lung caused by preceding influenza infection (Figure 2A). However, *Stat2*^−/−^ mice were rescued from this clearance defect (Figure 2A). To assess survival in the context of super-infection, adult female mice were subjected to influenza/MRSA super-infection using a dose of MRSA previously determined to be lethal to WT mice. Given the increased severity of influenza infection seen in females, mice were infected with a lower dose of influenza to minimize the confounding effects of lethal influenza infection. Under these conditions, *Stat2*^−/−^ mice showed significantly delayed mortality (Figure 2B). Next, we found that BAL fluid from both *Stat2*^−/−^ and WT mice had significantly greater numbers of neutrophils compared to macrophages or lymphocytes (Figure 2C). However, we observed no significant differences in neutrophils, macrophages, or lymphocytes between super-infected WT and *Stat2*^−/−^ mice (Figure 2C). Further, we found no differences in the levels of cytokines IL-1α, IL-1β, IL-6, TNFα, IL-12p40, IL-17A, IL-10, CXCL1, and GCSF between WT and *Stat2*^−/−^ mice (Figures 2D–F). However, the levels of IFNγ, IL-4 and IL-5 were elevated in *Stat2*^−/−^ mice during super-infection (Figure 2E). In agreement with similar cellularity and local production of pro-inflammatory cytokines, histological scoring showed no differences in perivascular inflammation (Figures 2G,H). These findings demonstrate that *Stat2*^−/−^ mice are rescued from impaired bacterial clearance in the lung during super-infection, which translated into a detectable survival advantage, but this was independent of any noticeable differences in lung inflammation.

We next addressed whether the increased bacterial clearance observed in *Stat2*^−/−^ mice was due to altered Type 17 immunity. Interestingly, numbers of IL-17 and IL-22 positive CD4 cells, and the levels of Type 17 cytokines (IL-17, IL-22, and IL-23) were not altered in *Stat2*^−/−^ mice compared to WT mice (Figures S1A–E). In further support of this finding, no differences in expression levels of the Th17 transcription factors *Rorc* and *Rora*, and Th17 immune mediated antimicrobial peptide (AMP) such as *Lcn2* and *Reg3b* production in the lungs were detected between *Stat2*^−/−^ and WT mice (data not shown). These data suggest that protection in *Stat2*^−/−^ mice is not mediated by rescue of Type 17 immunity.

### Rescue of bacterial clearance in *stat2^−/−^* mice is associated with M1 and M2 signature in infected lungs

**Figure 3:**
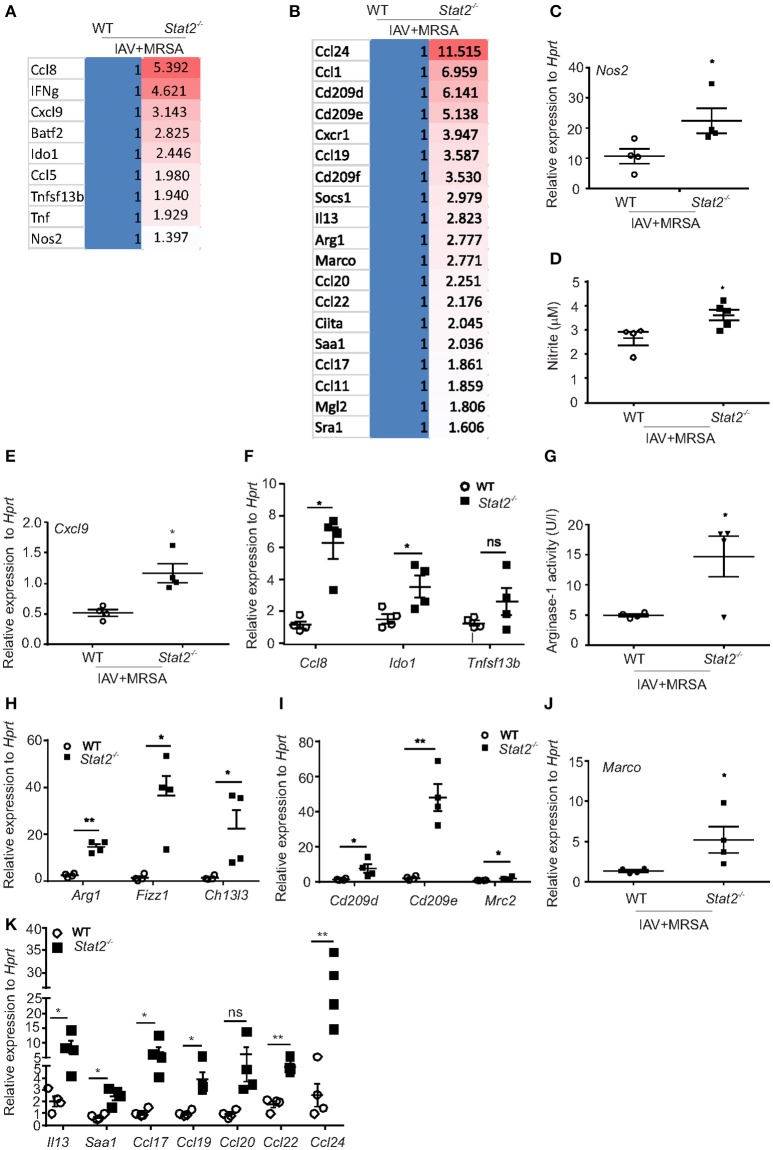
M1 and M2 macrophages are associated with increased bacterial clearance in *Stat2*^−/−^ during influenza-MRSA super-infection. WT or *Stat2*^−/−^ mice were infected with 100 PFU of influenza for 6 days then challenged with 5 × 10^7^ cfu of MRSA for one additional day. Gene expression analyses were measured in lung by RNAseq analysis. Heat-map representing RNA abundance associated with M1 **(A)** and M2 **(B)** macrophages from WT and *Stat2*^−/−^ mice, *N* = 4 per group. **(C)** *Nos2*, **(D)** Nitrite, **(E)** *Cxcl9*, **(F)** *Ccl8, Ido1, Tnfsf13b* were analyzed by RT-PCR, *N* = 4 per group. **(G)** Arginase-1 activity was determined from BAL samples from both WT and *Stat2*^−/−^ mice infected with influenza and MRSA super-infection, *N* = 4 per group. **(H)** *Arg1, Fizz1*, and *Chi3l3*, **(I)** *Cd209d, Cd209e* and *Mrc2* **(J)** *Marco* mRNA expression levels were determined by RT-PCR, *N* = 3–4 per group. **(K)** *Il13* and *Saa1, Ccl17, Ccl19, Ccl20, Ccl22*, and *Ccl24* mRNA expression levels were determined by RT-PCR, *N* = 4 per group. Representative data shown from three or more experiments. Data are represented as mean±SEM. Data analyzed using 2-tailed Student's *t*-test, ^*^p < 0.05, ^**^p < 0.01, ns, not significant.

Next, to determine the pathways associated with this increased bacterial clearance in *Stat2*^−/−^ mice, we performed RNA sequencing analyses. We found increased expression of Type 1 (IFNγ) and Type 2 (IL-4 and IL-13) cytokines in *Stat2*^−/−^ mice compared to WT (Figures 2E, 3A,B). In response to IFNγ stimulation, macrophages increase their production of nitric oxide and reactive oxygen intermediates to become M1 type macrophages, which exhibit proinflammatory and antibacterial activity (30, 31) Accordingly, we found increased RNA abundance of genes associated with the M1 phenotype including *Nos2, Tnfa, Tnfsf13b, Batf2, Ido1*, and chemokines such as *Cxcl9, Ccl5*, and *Ccl8* in the lungs of *Stat2*^−/−^ mice compared to WT during super-infection (Figure 3A).

In response to IL-4, IL-13, and glucocorticoids, macrophages express markers of the M2 phenotype, which is characterized by induction of Arg1, chitinase 3-like 3 (Ym), found in inflammatory zone-1 (Fizz1), resistin-like molecule (Relmα/Retnla), chemokines such as CCL17, CCL20, and CCL22, scavenger receptors (CD36, Macrophage receptor with collagenous structure or MARCO), and c-type lectin receptors or CLRs (CD209, Macrophage Galactose Lectin or MGL) (30, 32). Accordingly, we found increased RNA abundance of *Arg1, Socs1, Sra1, Saa1, Ciita, Marco, Mgl2, Cd209d, Cd209e, Cd209f, Ccl1, Ccl11, Ccl17, Ccl19, Ccl20, Ccl22, and Ccl24* genes in *Stat2*^−/−^ mice when compared to WT mice during super-infection (Figure 3B).

Next, we confirmed the observed RNA abundance of the classical M1 and M2 macrophage markers. We found increased gene expression of *Nos2* and Nitrite in *Stat2*^−/−^ mice compared to WT with super-infection (Figures 3C,D). IFNγ-inducible chemokines CXCL9, CXCL10, and CXCL11 are ligands for the receptor CXCR3, which is essential in the attraction of effector lymphocytes to inflammatory sites (33, 34). Accordingly, we found increased mRNA expression of *Cxcl9* in influenza/MRSA infected *Stat2*^−/−^ mice compared to WT mice (Figure 3E). Further, we found increased expression of M1 macrophage markers *Ccl8, Ido1*, and *Tnfsf13b in Stat2*^−/−^ mice during super-infection when compared to WT mice (Figure 3F). These data confirm the increased expression of genes associated with M1 macrophages in *Stat2*^−/−^ mice during influenza-bacterial super-infection.

Next, we found increased mRNA expression and activity levels of Arg1, mRNA expression of *Fizz, Chi3l3, Cd209d, Cd209e, Mrc2* (*Cd206*), *Marco, Il13, Saa1* (Serum amyloid A1 protein), *Ccl17, Ccl19, Ccl20, Ccl22*, and *Ccl24* in *Stat2*^−/−^ mice compared to WT mice during super-infection (Figures 3G–K). Together, these data show that in the absence of STAT2 signaling, both the M1 and M2 macrophage phenotypes are enhanced during super-infection.

IFNγ signals through the IFNGR1/ IFNGR2 complex and activates the transcription factor STAT1, thereby inducing ISGs. We found increased levels of IFNγ (Figure 2E) and IFNγ-induced ISGs (Figure 3E) in *Stat2*^−/−^ mice during influenza-bacterial super-infection. Next, we determined whether STAT1 is altered due to increased levels of IFNγ in *Stat2*^−/−^ mice during influenza-bacterial super-infection. As expected, we found increased STAT1 expression in *Stat2*^−/−^ mice when compared to WT mice during influenza-bacterial super-infection (Figure S2A).

IL-4 activates the STAT6 signaling pathway through activation of transcription factors PPARγ and PPARδ and drives M2 polarization (31, 35). Here, we tested whether STAT6 signaling is altered due to increased IL-4 levels in *Stat2*^−/−^ mice during influenza-bacterial super-infection. However, we found no differences in STAT6 expression levels in between WT and *Stat2*^−/−^ mice during influenza-bacterial super-infection (Figure S2B). Further, we measured the RNA expression of *Pparg* from WT and *Stat2*^−/−^ mice during influenza-MRSA super-infection. Interestingly, we found that *Pparg* expression was suppressed in *Stat2*^−/−^ mice during influenza-bacterial super-infection (Figure S2C). Together, these data suggest that in the absence of STAT2, there is alteration of other associated signaling pathways during super-infection.

**Figure 4:**
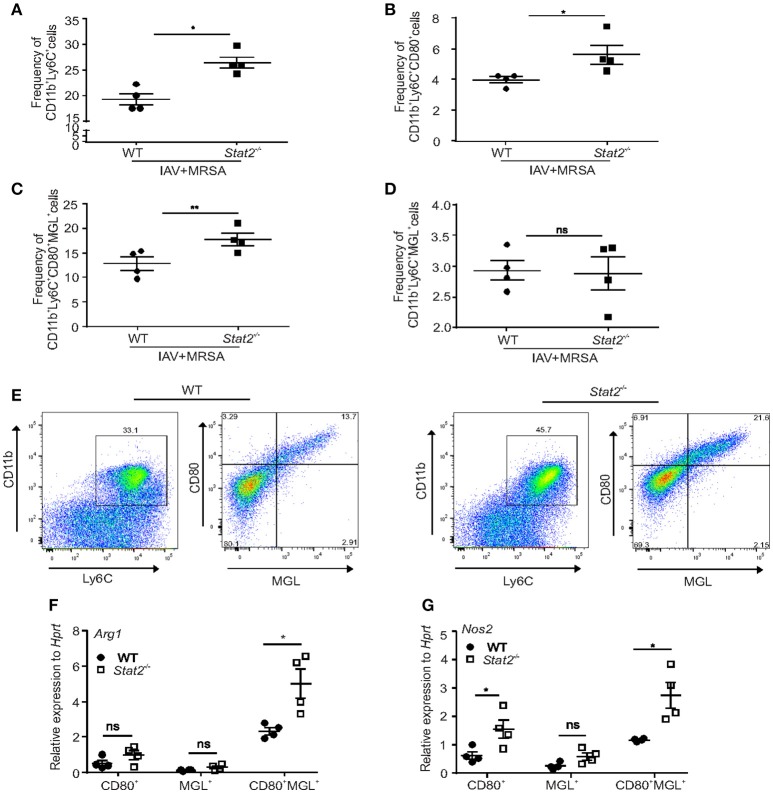
Increased frequency of M1/M2 macrophages in *Stat2*^−/−^ during influenza-MRSA super-infection. WT or *Stat2*^−/−^ mice were infected with 100 PFU of influenza for 6 days then challenged with 5 × 10^7^ cfu of MRSA for one additional day. **(A)** Frequency of CD11b^+^Ly6C^+^ cells, **(B)** CD11b^+^Ly6C^+^CD80^+^ cells, **(C)** CD11b^+^Ly6C^+^MGL^+^ cells **(D)** CD11b^+^Ly6C^+^CD80^+^MGL^+^ cells from lungs were determined from influenza and MRSA super infection by flow cytometry, *N* = 4 per group. Double positive CD11b^+^Ly6C^+^ cells were gated for CD80 and MGL to identify the CD11b^+^Ly6C^+^CD80^+^, CD11b^+^Ly6C^+^MGL^+^, CD11b^+^Ly6C^+^CD80^+^MGL^+^ cells. **(E)** The representative figures were shown. CD80^+^, MGL^+^, CD80^+^MGL^+^ cells were sorted from the lung using FACS and Arg1 **(F)** NOS2 **(G)** RNA expression was analyzed using RT-PCR. *N* = 4 per group. Data analyzed using 2-tailed Student's *t*-test, ^*^p < 0.05, ^**^p < 0.01, ns, not significant.

Next, we determined the frequency of M1 and M2 macrophages by using flow cytometry. CD80 and MGL (macrophage galactose lectin) have been shown to be markers for M1 and M2 macrophages, respectively (36, 37). We found a significant increase in the percentage of CD11b^+^Ly6C^+^ cells in influenza/MRSA infected *Stat2*^−/−^ lungs when compared to WT mice by flow cytometry (Figure 4A). Further, we found increased frequency of M1 (CD80^+^) and M1/M2 (CD80^+^MGL^+^) co-expressing macrophages in *Stat2*^−/−^ mice when compared to WT during influenza-bacterial super-infection (Figures 4B,C). Interestingly, we found no differences in M2 (MGL^+^) macrophages in between WT and *Stat2*^−/−^ mice in super-infection (Figure 4D). Further, we found higher frequency of M1/M2 co-expressing macrophages when compared to M1 or M2 populations (Figures 4B–E).

To then determine the specific macrophages involved in production of Arg1 and Nos2, we sorted M1, M2, and M1/M2 cells by using specific antibodies, and determined the RNA expression levels of *Nos2* and *Arg1* (Figures 4F,G). As expected, we found increased expression of *Nos2* in M1 and M1/M2 macrophages. Next, we found no differences in induction of *Arg1* in M1 and M2 macrophages. However, we found increased expression of *Arg1* in dual M1/M2 macrophages. These data suggest that these M1/M2 macrophages induce both Arg1 and Nos2 in influenza-bacterial super-infection. To further characterize specific Arg1^+^ and iNOS^+^ macrophage localization in the lung, we used an IHC technique using iNOS and Arg1 antibodies.

### Increased accumulation of M1 and M2 macrophages in *stat2^−/−^* mice is dependent on preceding influenza infection

**Figure 5:**
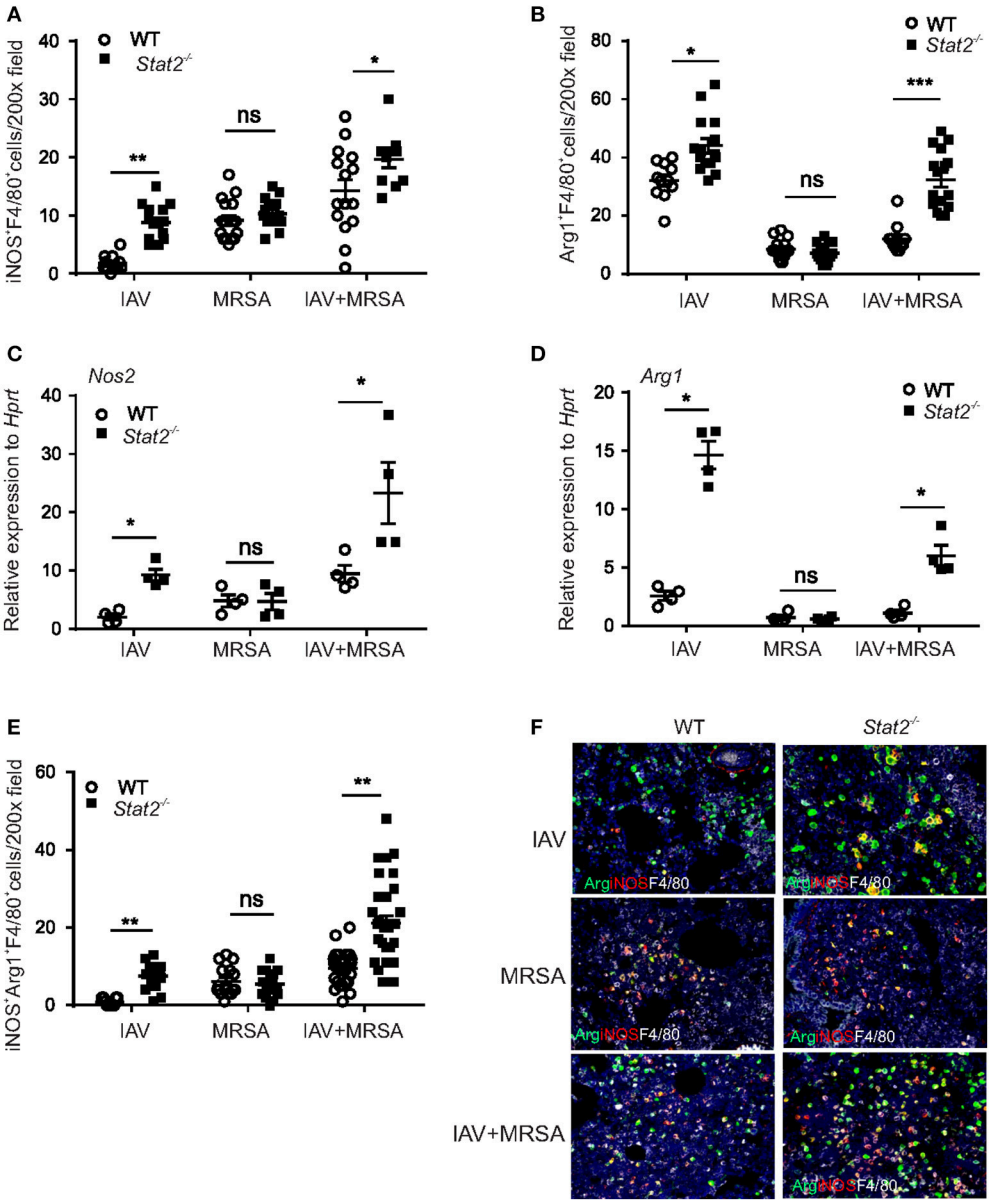
Increased M1 and M2 macrophage expression in *Stat2*^−/−^ is dependent on influenza but not MRSA infection. WT or *Stat2*^−/−^ mice were infected with 100 PFU of influenza for 6 days or 5 × 10^7^ cfu of MRSA for one day or super-infection as described in methods. **(A)** iNOS^+^F4/80^+^, *N* = 11–16 **(B)** Arg1^+^F4/80^+^ producing cells were determined from lungs by IHC, *N* = 11–16. **(C)** *Nos2*, **(D)** *Arg1* gene expression was analyzed from lungs by RT-PCR, *N* = 4 per group. **(E)** iNOS^+^Arg1^+^F4/80^+^ producing cells were determined from lung by immunohistochemistry, *N* = 11–32 per group. **(F)** Representative figures, × 200 magnification fields are shown. Data are represented as mean±SEM. Data analyzed using two-tailed Student's *t*-test, ^*^p < 0.05, ^**^p < 0.01, ^***^p < 0.001, ns, not significant.

To determine whether the increase in M1 and M2 macrophages is influenza or MRSA dependent, we infected WT or *Stat2*^−/−^ mice with influenza or MRSA or super-infection, and determined the number of iNOS^+^F4/80^+^ and Arg1^+^F4/80^+^ macrophages in the lung by IHC. We found increased numbers of iNOS^+^F4/80^+^ and Arg1^+^F4/80^+^ cells during influenza infection and super-infection in *Stat2*^−/−^ mice compared to WT mice (Figure 5A). In WT mice, we observed a higher number of iNOS^+^F4/80^+^ cells during MRSA and super-infection compared to influenza infection alone. In contrast, we observed a decrease in the number of Arg1^+^F4/80^+^ cells in MRSA and super-infection compared to WT mice infected with influenza alone (Figure 5B). Further, no differences were observed between WT and *Stat2*^−/−^ mice in the number of iNOS^+^F4/80^+^ and Arg1^+^F4/80^+^ cells during MRSA infection alone (Figures 5A,B). Next, we found increased gene expression of *Nos2* and *Arg1* in *Stat2*^−/−^ mice when compared to WT mice during influenza, but not MRSA infection (Figures 5C,D). These data indicate that influenza infection, not MRSA infection, is likely the cause of the increased number of Arg1^+^ macrophages seen in *Stat2*^−/−^ mice during super-infection.

The M1 and M2 macrophage phenotypes are thought to antagonize each other to establish inflammatory or anti-inflammatory responses (38). M1 macrophages induce L-hydroxy-arginine and citrulline through NOS2 induction, which is a potent inhibitor of arginase (39), to promote inflammation. Conversely, M2 macrophage induction of arginase antagonizes M1 markers to promote repair and resolution of infection (39). As we seen in flow cytometry data, we found an increased number of “dual function” iNOS^+^Arg1^+^F4/80^+^ cells during and super-infection in *Stat2*^−/−^ mice compared to WT mice (Figures 5E,F). However, no significant differences were observed in the number of dual function iNOS^+^Arg1^+^F4/80^+^ cells between WT and *Stat2*^−/−^ mice during MRSA infection (Figures 5E,F). Together, these data show that in the absence of STAT2 signaling, the increase in Type 1 and Type 2 cytokines during influenza infection creates a pulmonary environment that supports both M1 and M2 macrophage differentiation after super-infection. Unexpectedly and contrary to the idea that there is antagonism between M1 and M2 macrophages, we found increased accumulation of M1, M2 and M1/M2 co-expressing cells in the lungs of *Stat2*^−/−^ mice after super-infection.

### Dual function M1/M2 macrophages are required for control of bacterial burden in *stat2^−/−^* mice

**Figure 6:**
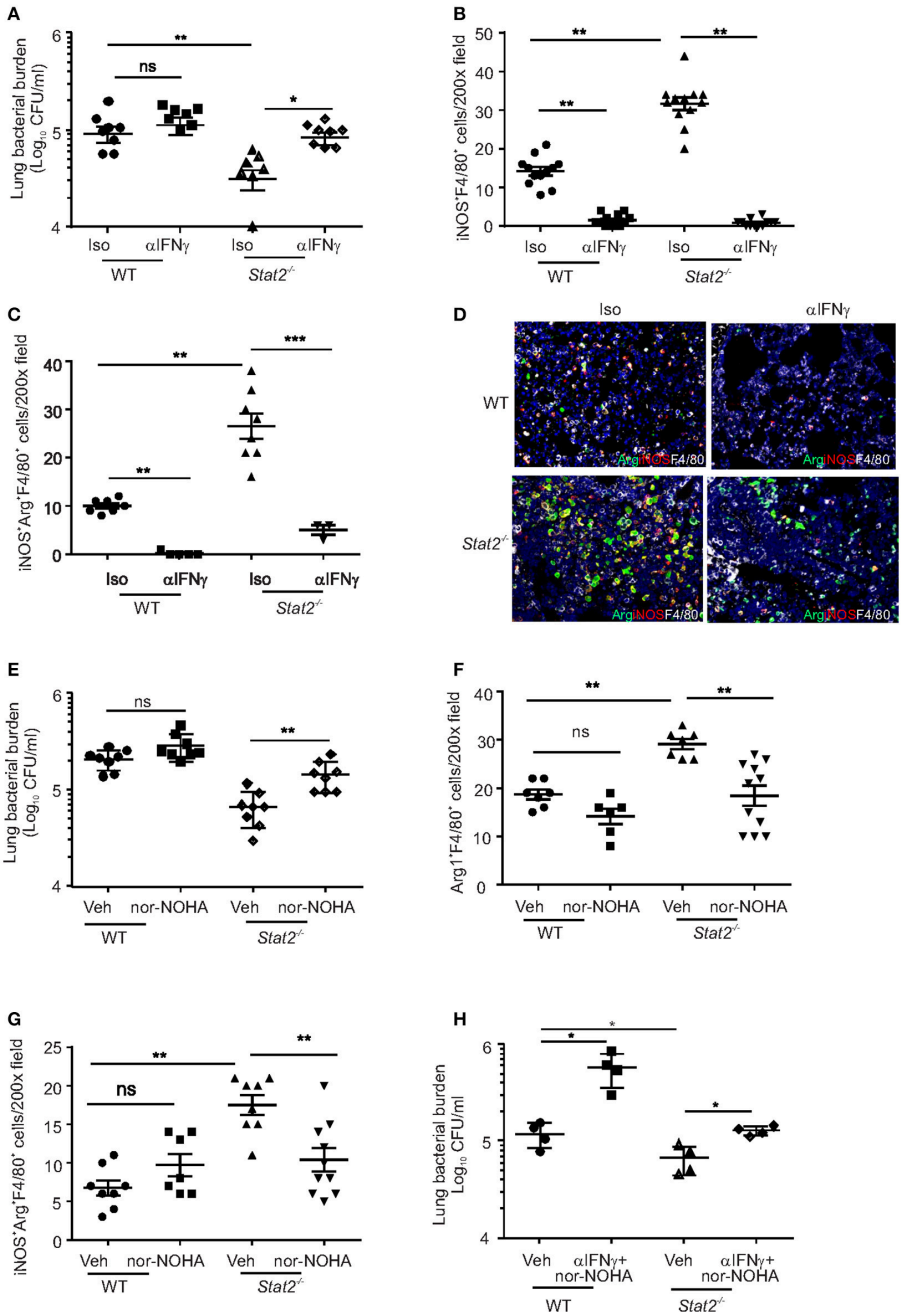
M1/M2 co-expressing macrophages are required for increased bacterial clearance in *Stat2*^−/−^ during influenza-MRSA super-infection. WT or *Stat2*^−/−^ mice were infected with 100 PFU of influenza for 6 days then challenged with 5 × 10^7^ cfu of MRSA for one additional day. Mice were treated with 300 μg of anti-IFNγ antibodies or 300 μg of rat IgG isotype controls as described in methods. **(A)** Bacterial burden was measured, *N* = 7–8 per group, **(B)** iNOS^+^F4/80^+^ producing cells, *N* = 12 per group **(C)** iNOS^+^Arg1^+^F4/80^+^ producing cells, *N* = 3–7 per group **(D)** were determined by IHC, representative figures x200 magnification fields are shown. WT or *Stat2*^−/−^ mice were infected with 100 PFU of influenza for 6 days then challenged with 5 × 10^7^ cfu of MRSA for one additional day. Mice were treated with Vehicle (DMSO) or N-^−^hydroxy-nor-L-arginine (nor-NOHA) as described in methods **(E)** Bacterial burden, *N* = 8 per group, **(F)** Arg^+^F4/80^+^ cells, *N* = 6–10 per group **(G)** iNOS^+^Arg^+^F4/80^+^ producing cells were determined, *N* = 7–10 per group. Representative pictures from two experiments are shown. **(G)** WT or *Stat2*^−/−^ mice were infected with 100 PFU of influenza for 6 days then challenged with 5 × 10^7^ cfu of MRSA for one additional day. Mice were treated with 300 μg of anti-IFNγ antibodies and N-^−^hydroxy-nor-L-arginine (nor-NOHA) or vehicle as described in methods **(H)** Bacterial burden was measured, *N* = 4 per group, Data are represented as mean±SEM. Data analyzed using 2-tailed Student's *t*-test or One way ANOVA followed by Bonferroni test for multiple comparisons, ^*^p < 0.05, ^**^p < 0.01, ^***^p < 0.001, ns, not significant.

We next examined whether M1 and/or M2 macrophages are required for the improved bacterial clearance in *Stat2*^−/−^ mice during super infection. We treated both influenza-infected WT and *Stat2*^−/−^ mice with anti-IFNγ, followed by super-infection with MRSA, and measured subsequent bacterial burden. We found that blocking IFNγ decreased bacterial clearance in *Stat2*^−/−^ mice (Figure 6A). IFNγ neutralization showed a trend toward decreased bacterial clearance in WT mice as well. Next, we found fewer iNOS^+^F4/80^+^ cells in anti-IFNγ-treated WT and *Stat2*^−/−^ mice compared to mice treated with isotype controls (Figure 6B). Further, we found a decreased number of dual function iNOS^+^Arg1^+^F4/80^+^ in mice treated with anti-IFNγ antibody (Figures 6C,D). However, consistent with the role of IFNγ as an inducer of the M1 phenotype, we found no significant differences in the number of total Arg1^+^F4/80^+^ cells with IFNγ neutralization (Figure S3A).

Next, we determined whether M2 macrophage activation was required to control MRSA during super-infection in *Stat2*^−/−^ super-infected mice. Arg1 was neutralized using nor-NOHA in both WT and *Stat2*^−/−^ mice subjected to influenza/MRSA super-infection. Inhibition of Arg1 during super-infection increased bacterial burden in *Stat2*^−/−^, but not WT mice (Figure 6E). Further, we observed a decrease in the number of Arg1^+^F4/80^+^ macrophages and dual function iNOS^+^Arg1^+^F4/80^+^ macrophages in *Stat2*^−/−^ mice treated with nor-NOHA when compared to *Stat2*^−/−^ mice treated with vehicle (Figures 6F,G). Interestingly, we found increased numbers of iNOS^+^F4/80^+^ macrophages in both WT and *Stat2*^−/−^ mice upon nor-NOHA treatment during influenza-bacterial infection (Figure S3B). Despite the presence of M1 macrophages during Arg1/M2 blockade and M2 macrophages during IFNγ/M1 blockade, bacterial control was suppressed in *Stat2*^−/−^ mice during super-infection. These data suggest that neither M1 nor M2 macrophages alone are as proficient at MRSA clearance. We then inhibited both IFNγ and Arg1 simultaneously during super-infection in WT and *Stat2*^−/−^ mice (Figure 6H). Inhibition of both M1 and M2 pathways increased bacterial burden in both WT and *Stat2*^−/−^ mice. These data together suggest that activation of M1/M2 co-expressing macrophages is required for increased bacterial clearance during super-infection in *Stat2*^−/−^ mice.

### STAT2 signaling negatively regulates macrophage bacterial uptake and killing

**Figure 7:**
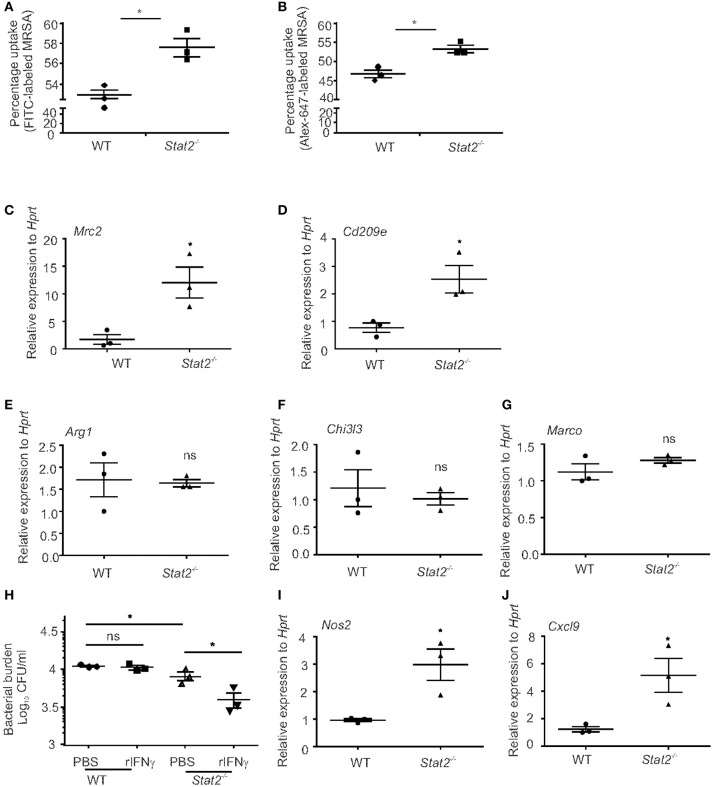
Increased bacterial uptake and killing efficiency in BMDMs from *Stat2*^−/−^ mice BMDMs were generated and infected with FITC or Alex-647-labeled MRSA (10 MOI) for 30 min, washed, fixed with 1% formaldehyde and the number of FITC^+^ **(A)** or Alex-647^+^ **(B)** BMDMs were determined by Flow cytometry. **(C)** *Mrc2* and **(D)** *Cd209e*, **(E)** *Arg1*, **(F)** *Chi3l3*, **(G)** *Marco* gene expression levels were measured by RT-PCR. **(H)** BMDMs were generated, treated with recombinant IFNγ (10 μg/ml) for 24 h, and infected with MRSA (10 MOI) and bacterial killing was determined. **(I)** *Nos2* and **(J)** *Cxcl9* gene expression levels were measured from naïve BMDMs, *N* = triplicates per treatment. Representative pictures from two experiments are shown. Data are represented as mean±SEM. Data analyzed using 2-tailed Student's *t*-test, ^*^p < 0.05, ns, not significant.

We then examined bacterial uptake and killing by macrophages from WT and *Stat2*^−/−^ mice. BMDMs isolated from WT and *Stat2*^−/−^ mice were incubated with fluorescent-labeled MRSA for 30 min. Bacterial uptake percentage was determined by the number of FITC^+^ BMDMs by flow cytometry. It has been shown that the FITC dye can be quenched in response to pH changes in endosomes (40, 41). Therefore, we compared phagocytosis by using FITC or Alexa Fluor-647-labeled bacteria. We found increased bacterial uptake in *Stat2*^−/−^ mice compared to WT mice in both FITC or Alexa Fluor-647-labeled MRSA (Figures 7A,B). These data show that macrophages from *Stat2*^−/−^ mice can bind and take up more bacteria than WT mice. Further, under basal conditions we found increased mRNA expression of *Mrc2* and *Cd209e* in *Stat2*^−/−^ BMDMs compared to WT BMDMs (Figures 7C,D). However, no significant differences in the expression levels of *Arg1, Chi3l3*, and *Marco* were observed (Figures 7E–G). These results suggest that increased expression of the M2 phenotype-associated receptors MRC2 and CD209 is likely to be a mechanism involved in increased binding ability in BMDMs from *Stat2*^−/−^ mice.

To test the ability of these BMDMs to kill MRSA, we performed a bacterial killing assay using BMDMs treated with mouse IFNγ. We found that *Stat2*^−/−^ BMDMs have increased killing ability at baseline and following IFNγ treatment when compared to WT BMDMs (Figure 7H). However, in BMDMs from WT mice, IFNγ treatment had no effect on bacterial killing (Figure 7H). Next, we found that macrophages from *Stat2*^−/−^ mice had increased expression of *Nos2* and *Cxcl9* compared to WT mice (Figures 7I,J). These data suggest that macrophages from *Stat2*^−/−^ mice have an enhanced ability to kill bacteria due to increased activation and nitric oxide production, and have the potential to attract CXCR3^+^ effector lymphocytes that can amplify M1 activation. These results together show that STAT2 modulates the expression of CLRs and iNOS production by macrophages, and thus negatively impacts their protective functions during super-infection.

### Contribution of hematopoietic or stromal cell compartments in *stat2^−/−^* mediates improves MRSA clearance in super-infection

**Figure 8:**
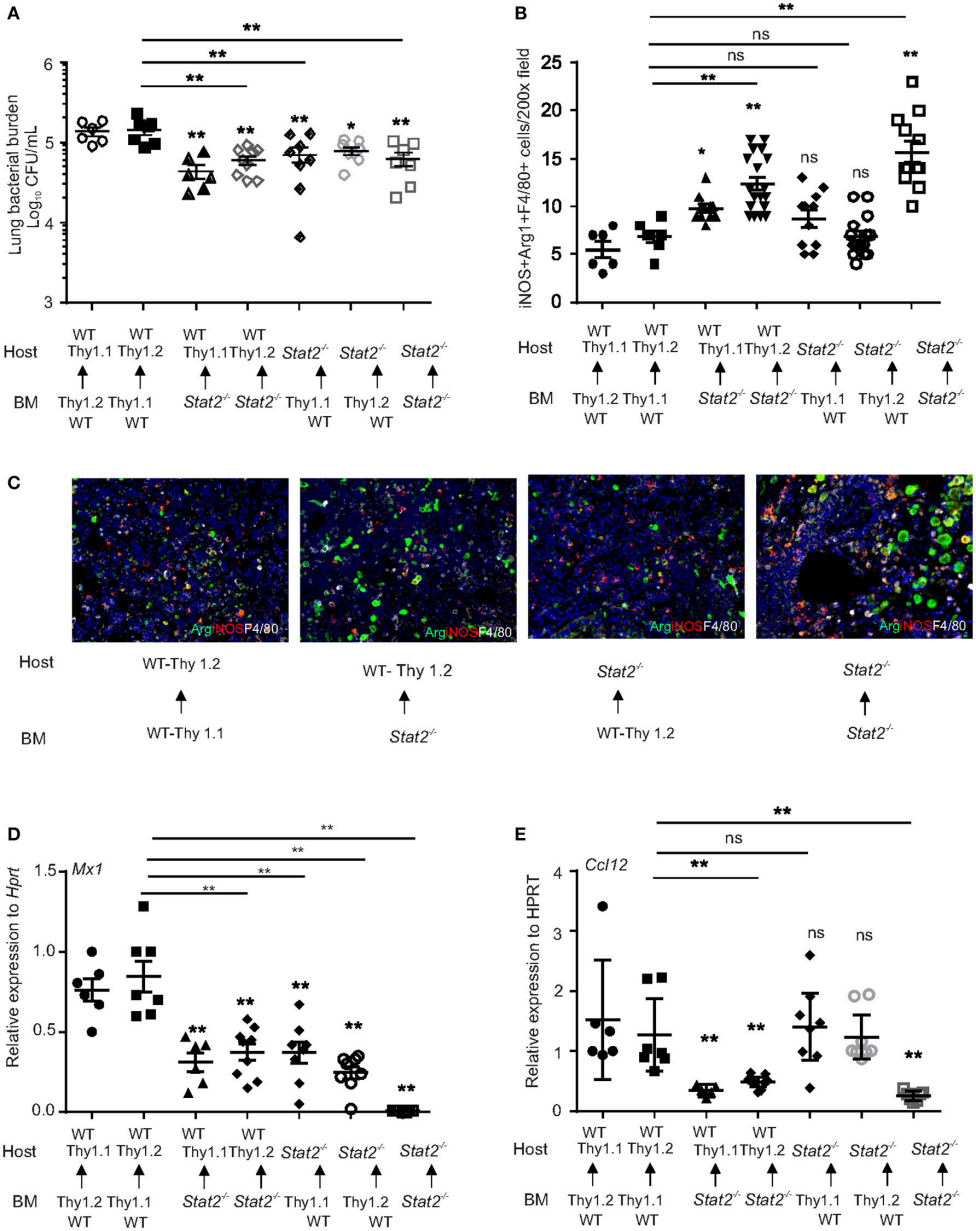
Increased bacterial control in cells from hematopoietic or non-hematopoietic compartments of *Stat2*^−/−^ mice. WT BMC (Thy1.1 host, Thy 1.2 BM or Thy 1.2 host, Thy 1.1 BM), *Stat2*^−/−^ BMC (*Stat2*^−/−^ host, *Stat2*^−/−^ BM), Hematopoietic *Stat2*^−/−^ BMC mice (Thy 1.1 or Thy 1.2 host, *Stat2*^−/−^ BM), non- hematopoietic *Stat2*^−/−^ BMC (*Stat2*^−/−^ host, Thy 1.1 or Thy 1.2 BM) were generated as described in methods. These mice were infected with 100 PFU of influenza for 6 days then challenged with 5 × 10^7^ cfu of MRSA for one additional day. **(A)** Mice were sacrificed and right upper lung lobes were homogenized in PBS and bacterial burden was measured. **(B)** iNOS^+^Arg1^+^F4/80^+^ producing cells were determined from lung by immunohistochemistry, *N* = 6–19 per group. **(C)** Representative figures, × 200 magnification fields are shown. **(D)** *Mx1*, **(E)** *Ccl12* mRNA expression was measured, *N* = 6–9 per group. Data are represented as mean±SEM. Data analyzed using One way ANOVA followed by Bonferroni test for multiple comparisons, ^*^p < 0.05, ^**^p < 0.01, ns, not significant.

To further confirm that increased bacterial control in *Stat2*^−/−^ mice during influenza-bacterial super infection is due to M1/M2 co-expressing macrophage activation, we generated bone marrow chimeric (BMC) mice. We transferred hematopoietic cells from WT or *Stat2*^−/−^ mice to irradiated WT or STAT2 mice and vice versa. We used WT C57BL/6 mice with Thy1.1 or Thy 1.2 markers and *Stat2*^−/−^ mice bred on a Thy1.2 background. After 9 weeks of bone marrow reconstitution, we infected mice with both influenza and MRSA. Consistent with our previous data, we found higher lung bacterial burden in WT BMC mice (WT host/WT BM) than *Stat2*^−/−^ mice (*Stat2*^−/−^ host/*Stat2*^−/−^ BM) during influenza-bacterial super-infection (Figure 8A). As expected, we found decreased bacterial burden in hematopoietic *Stat2*^−/−^ BMC mice (WT host/*Stat2*^−/−^ BM) compared to WT BMC mice (WT host/ WT BM) during super-infection (Figure 8A). Interestingly, we also found decreased bacterial burden in non-hematopoietic *Stat2*^−/−^ BMC (*Stat2*^−/−^ host/WT BM) compared to WT BMC mice (WT host/ WT BM) during super-infection (Figure 8A).

Next, we found increased expression of iNOS^+^Arg1^+^ macrophages in hematopoietic *Stat2*^−/−^ BMC (WT host/*Stat2*^−/−^ BM) and *Stat2*^−/−^ BMC mice (*Stat2*^−/−^ host/*Stat2*^−/−^ BM), suggesting that hematopoietic cells are involved in bacterial control in *Stat2*^−/−^ mice during influenza-MRSA super-infection (Figures 8B,C). Also, no significant differences were observed in the expression of iNOS^+^Arg1^+^ macrophages between WT BMC mice (WT host/WT BM) and non-hematopoietic *Stat2*^−/−^ BMC mice (*Stat2*^−/−^ host/WT BM). These data suggest that iNOS^+^Arg1^+^ macrophages mediate bacterial control in hematopoietic *Stat2*^−/−^ BMC (WT host/ *Stat2*^−/−^ BM) and *Stat2*^−/−^ BMC mice (*Stat2*^−/−^ host/*Stat2*^−/−^ BM) during influenza-MRSA super-infection. However, we found increased bacterial clearance in non-hematopoietic *Stat2*^−/−^ BMC mice (*Stat2*^−/−^ host/WT BM) during influenza-MRSA super-infection.

Type I and type II interferons induce interferon stimulated genes (ISGs) to establish anti-viral response responses (42–44). Based on RNAseq data and RT-PCR analysis, we identified that expression of type I IFN-induced genes such as *Mx1, Lhx2*, and *CCL12* was suppressed in *Stat2*^−/−^ mice during influenza-MRSA super-infection (Figures S4A–D). However, the type II IFN-induced chemokine *CXCL9* was not suppressed during influenza-MRSA super-infection (Figure S4E). Further, *Mx1* and *Ccl12* gene expression was suppressed in bone marrow dendritic cells (BMDCs) from *Stat2*^−/−^ mice stimulated with IFNβ compared to BMDCs from WT mice (Figures S4F,G). These data confirm that type I IFN, but not type II IFN responses are significantly attenuated in the lungs of *Stat2*^−/−^ mice during influenza infection.

To determine if ISG expression in *Stat2*^−/−^ BMC mice was suppressed, we measured the expression of *Mx1* and *Ccl12*. We found decreased expression of *Mx1* in *Stat2*^−/−^ BMC (*Stat2*^−/−^ host/*Stat2*^−/−^ BM), hematopoietic *Stat2*^−/−^ BMC (WT host/ *Stat2*^−/−^ BM), and non-hematopoietic *Stat2*^−/−^ BMC mice (*Stat2*^−/−^ host/WT BM) compared to WT BMC mice (WT host/WT BM) (Figure 8D). The expression of *Mx1* was further suppressed in *Stat2*^−/−^ BMC mice (*Stat2*^−/−^ host/*Stat2*^−/−^ BM) when compared to hematopoietic *Stat2*^−/−^ BMC (WT host/ *Stat2*^−/−^ BM) and non-hematopoietic *Stat2*^−/−^ BMC mice (*Stat2*^−/−^ host/WT BM) (Figure 8D). These data suggest that both hematopoietic and non-hematopoietic cells are involved in the expression of *Mx1* in response to influenza-bacterial super-infection.

Next, we determined the expression of *Ccl12* in the BMC mice. We found decreased expression of *Ccl12* in *Stat2*^−/−^ BMC (*Stat2*^−/−^ host/*Stat2*^−/−^ BM) and hematopoietic *Stat2*^−/−^ BMC mice (WT host/ *Stat2*^−/−^ BM) as compared to WT BMC (WT host/WT BM) (Figure 8E). However, the expression of *Ccl12* was not suppressed in *Stat2*^−/−^ non-hematopoietic BMC mice (*Stat2*^−/−^ host/WT BM) (Figure 8E). These data suggest that STAT2 signaling in hematopoietic cells is crucial in induction of CCL12 during influenza-bacterial super-infection. Further, these data suggest that the absence of stromal cell STAT2 resulted in decreased ISG expression and improved bacterial control in *Stat2*^−/−^ non-hematopoietic BMC mice (*Stat2*^−/−^ host/WT BM) during influenza-bacterial super-infection.

Next, we determined the influenza viral burden in the BMC mice. We found higher viral burden in *Stat2*^−/−^ BMC (*Stat2*^−/−^ host/*Stat2*^−/−^ BM) and non-hematopoietic *Stat2*^−/−^ BMC mice (*Stat2*^−/−^ host/WT BM) when compared to WT BMC (WT host/WT BM) and hematopoietic *Stat2*^−/−^ BMC (WT host/ *Stat2*^−/−^ BM) (Figure S5). These data suggest that non-hematopoietic cells are involved in STAT2-mediated viral control during influenza-MRSA super-infection.

## Discussion

During influenza infection, epithelial cells, macrophages and dendritic cells all induce type I and type III IFNs, proinflammatory cytokines and chemokines (2). Type I IFN signaling mediates lung pathology and infiltration of granulocytes during influenza infection (7). Absence of STAT1 or STAT2 signaling compromises viral control and survival in mice (9). In the current study, we have demonstrated that in the absence of STAT2 signaling the type I IFN response is impaired. This was associated with increased viral burden, inflammation, and pathology in the lungs during influenza infection. These results show that STAT2-dependent signaling is crucial in controlling influenza burden and inflammatory immune responses during primary influenza infection. Consistent with these findings, elevated levels of type I IFNs during influenza infection correlates with disease severity in outbred mice (45).

Influenza-associated secondary bacterial pneumonia is a serious complication of influenza infection (10, 11, 46). Reducing morbidity and mortality requires insight into the immune mechanisms that alter susceptibility to secondary bacterial infection. In this study, we show that STAT2 deficiency improves survival and rescues the impairment of bacterial clearance from the lung otherwise observed during influenza-bacterial super-infection. Further, we have identified increased accumulation of M1, M2 and M1/M2 co-expressing macrophages by influenza-MRSA super-infection in the setting of STAT2 deficiency as a novel mechanism that mediates this protection.

We have previously demonstrated that influenza-induced type I IFN-mediated suppression of Type 17 responses to both Gram (+) (*S. aureus*) and Gram (−) bacteria (*Pseudomonas aeruginosa, Escherichia coli*) during influenza-bacterial super-infection (13, 14). In these studies, loss of Type 17 immune responses was associated with exacerbation of secondary bacterial challenge during influenza infection. Further, we have also shown increased bacterial control in *Ifnar*^−/−^ mice during super-infection, suggesting that influenza-induced type I IFN is a critical mediator of antibacterial immune suppression (13). Also, we have recently shown increased Type 17 immunity in STAT1^−/−^ mice during super-infection (17). Therefore, we hypothesized that STAT2 deficiency would rescue suppression of Type 17 responses during influenza infection. Surprisingly, we found that the increased bacterial clearance we observed in the absence of STAT2 was not due to rescue of the Type 17 immune response. These data suggest that Type 17 immunity is predominantly regulated by STAT1 and not STAT2 during super-infection. This finding prompted further investigation into the mechanism of protection in *Stat2*^−/−^ mice.

Since Type 17 responses were not associated with the observed phenotype, we next explored other possible mechanisms involved in bacterial control. In our study, we found no differences in neutrophils, macrophages and lymphocyte numbers in BAL of WT and *Stat2*^−/−^ mice during influenza-bacterial super-infection. A possible role for neutrophils in bacterial killing exists, as shown by a trend toward increased neutrophil numbers in BAL from *Stat2*^−/−^ mice. However, based on RNAseq data analysis, we found increased RNA abundance of M1 and M2 macrophage markers, and further characterized the RNA expression, frequency and immunolocalization of these cells. We then determined the role of these macrophages in bacterial control in *Stat2*^−/−^ mice during influenza-bacterial super-infection.

M1 macrophages are known to be involved in pathogen defense and inflammation, whereas M2 macrophages are thought to have a suppressive or regulatory role during inflammation (30, 47). In the current study, we found the majority of CD11b^+^Ly6C^+^ macrophages are CD80^+^/MGL^+^ (M1/M2) positive. Also, we found increased frequency of M1 (CD80^+^), but not M2 (MGL^+^) single positive cells in *Stat2*^−/−^ mice during influenza-bacterial super-infection. Further, these M1/M2 cells had increased expression levels of both *Arg1* and *Nos2* in *Stat2*^−/−^ mice during influenza-bacterial super-infection. We then confirmed these flow cytometry findings by IHC and observed an increase in Arg1^+^iNOS^+^F4/80^+^ cells in *Stat2*^−/−^ mice. These data confirm the ability of STAT2 to regulate macrophage phenotype during pneumonia, and identify an M1/M2 dual phenotype macrophage population in the context of influenza-associated secondary bacterial infection.

Further, in accordance with increased levels of IFNγ and ISGs, we found increased *Stat1* expression levels in *Stat2*^−/−^ mice when compared to WT mice. This suggests that in the absence of STAT2 signaling, other pathways are activated as a compensatory effect. However, *Stat6* expression is not altered in the absence of STAT2 in super-infection. IFNγ is known to suppress expression of the nuclear receptor PPARγ (48, 49). We also found suppression of *Pparg* expression in *Stat2*^−/−^ mice during influenza-bacterial super-infection. Alterations in IFNγ and PPARγ signaling may be a possible mechanism by which M1/M2 macrophage populations are altered in *Stat2*^−/−^ mice in super-infection.

In *Stat2*^−/−^ mice, iNOS^+^ macrophages were elevated during super-infection. This contrasted with WT mice in which iNOS^+^ macrophages were only induced in the setting of bacterial challenge. In contrast, Arg1^+^ macrophages were decreased during both MRSA and super-infection in WT mice. However, in *Stat2*^−/−^ mice the Arg1^+^ cells were increased in influenza-bacterial super-infection, but not MRSA infection, suggesting that the M2 induction during super-infection is driven by influenza infection. Chen et al. (15) correlated an increased number of M2 macrophages during influenza infection with suppression of the protective immune response to bacterial super-infection (50). In contrast, one study has shown that *S. aureus* priming increased M2 macrophages and anti-inflammatory responses to influenza challenge (51). In the current study, we found an increased number of Arg1^+^ macrophages associated with increased bacterial control in *Stat2*^−/−^ mice during super-infection, and that WT mice suffered from impaired bacterial clearance despite the presence of Arg1^+^ at a level similar to that seen during MRSA infection alone. Also, we found that Arg1- and iNOS-expressing dual function macrophages were significantly higher in *Stat2*^−/−^ mice. The presence of M1/M2 co-expressing macrophages has recently been described in response to *Toxoplasma gondii* infection (52), but to our knowledge is a novel finding in pulmonary host defense.

Promotion of bacterial killing by IFNγ is well established for intracellular pathogens such as *Mycobacterium tuberculosis* and *Toxoplasma gondii*. However, information regarding its role in bacterial killing of *S. aureus* is limited. Studies have shown that influenza-induced IFNγ inhibits pneumococcal control during super-infection (53). In this study, we found a trend in increased bacterial burden in IFNγ neutralization in WT mice during super-infection. However, no differences were observed in WT BMDMs in bacterial killing in response to IFNγ treatment. Observed discrepancies in the role of IFNγ during super-infection might be due to differences in the dose and the strain of bacteria. We found that improved bacterial control seen during influenza-bacterial super-infection in *Stat2*^−/−^ mice was lost upon neutralization of IFNγ. This was associated with a loss of iNOS^+^ and iNOS^+^Arg1^+^ dual-function cells, suggesting that increased IFNγ in *Stat2*^−/−^ mice during super-infection mediates the protective phenotype by driving induction of M1-polarized macrophages. However, it is known that IFNγ increases neutrophil nitrite production and increases phagocytosis (54–56). It is also possible that other reactive oxygen species are involved in increased IFNγ-mediated bacterial clearance in *Stat2*^−/−^ mice during super-infection. Similarly, we found that neutralization of Arg1 decreased bacterial clearance in *Stat2*^−/−^ mice during super-infection in association with an attenuated number of iNOS^+^Arg1^+^ dual function macrophages. Ultimately, bacterial control in *Stat2*^−/−^ mice during super-infection was compromised when the number of iNOS^+^Arg1^+^ dual function macrophages was diminished. Further, we confirmed this by decreased bacterial clearance upon both Arg1 and IFNγ neutralization in both WT and *Stat2*^−/−^ mice.

Further, BMC studies showed increased bacterial control in both *Stat2*^−/−^ BMC and *Stat2*^−/−^ hematopoietic BMC mice during influenza-bacterial infection, confirming the role of macrophage STAT2 in suppressing bacterial control during influenza-bacterial super-infection. However, bacterial control in non-hematopoietic *Stat2*^−/−^ mice is likely due to altered macrophage phenotype, as influenza-induced ISGs are reduced in these mice. Further, increased influenza viral burden in non-hematopoietic *Stat2*^−/−^ mice indicated a role for stromal STAT2 signaling in inducing ISGs. Collectively, these data indicate a cell intrinsic role for STAT2 signaling in macrophages and an accessory role for stromal STAT2 signaling via regulation of ISG expression.

In addition to quantitative differences in critical macrophage phenotypes, we have demonstrated that macrophages from *Stat2*^−/−^ mice showed important qualitative differences compared to WT cells. We demonstrated that BMDM from *Stat2*^−/−^ mice had improved MRSA uptake at baseline. This increased efficiency was unaffected by any infection challenge. Increased uptake was associated with expression of multiple C-type lectin receptors (CLRs), which are primarily expressed in monocytes in the lung, and are involved in phagocytosis of a variety of pulmonary pathogens (57, 58). However, these receptors favor the entry of influenza by acting as a receptor for viral attachment (59, 60). In this study, we found increased expression of CLRs and increased phagocytic ability in BMDMs from *Stat2*^−/−^ mice. These results suggest that, even though these receptors favor influenza infection, they may help control secondary bacterial infection in *Stat2*^−/−^ mice by improving bacterial uptake. In addition to increased bacterial uptake, BMDM from *Stat2*^−/−^ mice displayed increased bacterial killing compared to WT mice upon IFNγ treatment. Naive BMDMs from *Stat2*^−/−^ mice also displayed increased *Nos2* and *Cxcl9* expression compared to cells from WT mice, suggesting that *Stat2*^−/−^ macrophages have inherently increased expression of *Nos2* and enhanced bacterial killing ability compared to WT macrophages.

In summary, we have shown that STAT2 signaling decreases influenza viral burden and inflammatory immune responses during influenza infection, at the cost of inhibiting bacterial control during subsequent bacterial challenge by suppressing a distinct M1/M2 dual function macrophage population during influenza-bacterial super-infection. Together our data show a novel role of influenza induced type I IFN-mediated STAT2 signaling in inhibiting bacterial control through suppression of macrophage activity during influenza and influenza/MRSA super-infection. STAT2 and dual function M1/M2 macrophage activation may be a potential target for the treatment or prevention of influenza-bacterial super-infection.

## Ethics statement

All mouse experiments were approved by the University of Pittsburgh IACUC, Protocol #: 17071194; PHS Assurance Number: D16-00118.

## Author contributions

RG, BL, JR-M, and JA designed the experiments. RG, BL, KM, HR, KR, SM, MC, PS, RE, MM, KMR, and JR-M performed the experiments. RG, BL, JR-M, and JA performed the analyses. RG, BL, HR, MM, KMR, JR-M, and JA drafted and edited the manuscript.

### Conflict of interest statement

The authors declare that the research was conducted in the absence of any commercial or financial relationships that could be construed as a potential conflict of interest.

## Supplementary material

FIGURE S1 No differences in Th17 response between WT and *Stat2*^−/−^ mice following MRSA, and influenza-MRSA super-infection. WT or *Stat2*^−/−^ mice were infected with 100 PFU of influenza A/PR/8/H1N1 or PBS by oropharyngeal aspiration for 6 days then challenged with 5 × 10^7^ cfu of MRSA or PBS by oropharyngeal aspiration for one additional day. Frequency of **(A)** IL-17^+^ or **(B)** IL-22^+^ cells were determined by flow cytometry. The percentage of IL-17^+^ and IL-22^+^ cells were calculated from the percentage of CD3^+^CD4^+^ T cells. WT or *Stat2*^−/−^ mice were infected with influenza-MRSA as described above. Lung lobes were homogenized with PBS, and the levels of (**C**) IL-23, (**D**) IL-17 were measured by Luminex analysis, and **(E)** IL-22 levels measured by ELISA. Data are represented as mean ± SEM. Data analyzed using two-tailed Student's *t*-test, ns, not significant.

FIGURE S2 *Stat2*^−/−^ mice have altered other STAT1 and STA6 signaling pathways during influenza-bacterial super-infection. WT or *Stat2*^−/−^ mice were infected with either 100 PFU of influenza A PR/8/H1N1 or influenza for 6 days and then challenged with 5 × 10^7^ cfu of MRSA by oropharyngeal aspiration for one additional day, **(A)** *Stat1*, **(B)** *Stat6*, **(C)** *Pparg* gene expression relative to *Hprt* was analyzed by RT-PCR. *N* = 4 per group. Data are represented as mean ± SEM. Data analyzed using two-tailed Student's *t*-test, ^^*^^*p* ≤ 0.05, ns, not significant.

FIGURE S3 M1 and M2 macrophages are altered in *Stat2*^−/−^ mice in response to Arg1 and IFNγ neutralization during influenza-MRSA super-infection. WT or *Stat2*^−/−^ mice were infected with 100 PFU of influenza for 6 days then challenged with 5 × 10^7^ cfu of MRSA for one additional day. Mice were treated with 300 μg of anti-IFNγ antibodies or 300 μg of rat IgG isotype controls as described in methods. **(A)** Arg1^+^F4/80+ producing cells, were determined by IHC, *N* = 4–13 per group. WT or *Stat2*^−/−^ mice were infected with 100 PFU of influenza for 6 days then challenged with 5 × 10^7^ cfu of MRSA for one additional day. Mice were treated with Vehicle (DMSO) or N-hydroxy-nor-L-arginine (nor-NOHA) as described in methods. **(B)** iNOS+F4/80+ cells, *N* = 6–9 per group. Data are represented as mean ± SEM. Data analyzed using two-tailed Student's *t*-test, ^*^*p* ≤ 0.05, ^**^*p* ≤ 0.01, ns, not significant.

FIGURE S4 *Stat2*^−/−^ mice have deficient type I IFN responses with preserved type II IFN responses. **(A)** WT and *Stat2*^−/−^ male 6–8 weeks mice were infected with 100 PFU of influenza A/PR/8/H1N1 by oropharyngeal aspiration for 6 days then challenged with 5 × 10^7^ cfu of MRSA by oropharyngeal aspiration for one additional day. Overall gene expression was measured in lung by RNAseq analysis, Heat-map representing the fold induction of Interferon stimulated genes (ISGs) from WT and *Stat2*^−/−^ mice following influenza-MRSA super-infection, *N* = 4 per group. WT or *Stat2*^−/−^ mice were infected with either 100 PFU of influenza A PR/8/H1N1 or influenza for 6 days and then challenged with 5 × 10^7^ cfu of MRSA by oropharyngeal aspiration for one additional day, **(B)** *Mx1*, **(C)** *Lhx2*, and **(D)** *Ccl12*, **(E)** *Cxcl9* gene expression relative to *Hprt* was analyzed by RT-PCR. *N* = 3–4 per group. Bone marrow-derived dendritic cells (1 × 10^6^) were cultured *in vitro* and stimulated *in vitro* with IFNβ (6.5 units/mL) or IFNγ (20 ng/mL) for 24 h, **(F)** *Mx1*, **(G)** *Ccl12* gene expression in cell RNA was analyzed by qRT-PCR. The cells were treated in triplicates. Each data point from panel F and G were the number of replicates per treatment. Data are represented as mean ± SEM. Data analyzed using two-tailed Student's *t*-test, ^*^*p* ≤ 0.05, ^**^*p* ≤ 0.01, ns, not significant.

FIGURE S5 Increased influenza burden in cells from non-hematopoietic compartments of *Stat2*^−/−^ mice. WT BMC (Thy1.1 host, Thy 1.2 BM or Thy 1.2 host, Thy 1.1 BM), *Stat2*^−/−^ BMC (*Stat2*^−/−^ host, *Stat2*^−/−^ BM), Hematopoietic *Stat2*^−/−^ BMC mice (Thy 1.1 or Thy 1.2 host, *Stat2*^−/−^ BM), non-hematopoietic *Stat2*^−/−^ BMC (*Stat2*^−/−^ host, Thy 1.1 or Thy 1.2 BM) were generated as described in methods. These mice were infected with 100 PFU of influenza for 6 days then challenged with 5 × 10^7^ cfu of MRSA for one additional day. Mice were sacrificed and RNA expression of Influenza M protein was measured from the lungs by PCR, *N* = 6–9 per group. Data are represented as mean ± SEM. Data analyzed using One way ANOVA followed by Bonferroni test for multiple comparisons, ^**^*p* ≤ 0.01, ns, not significant.
