# Supplementary figures and images for: STAT2 Signaling Regulates Macrophage Phenotype During Influenza and Bacterial Super-Infection
Source: Front Immunol. 2018 Sep 25;9:2151. doi: 10.3389/fimmu.2018.02151 (PMC6178135; doi:10.3389/fimmu.2018.02151)

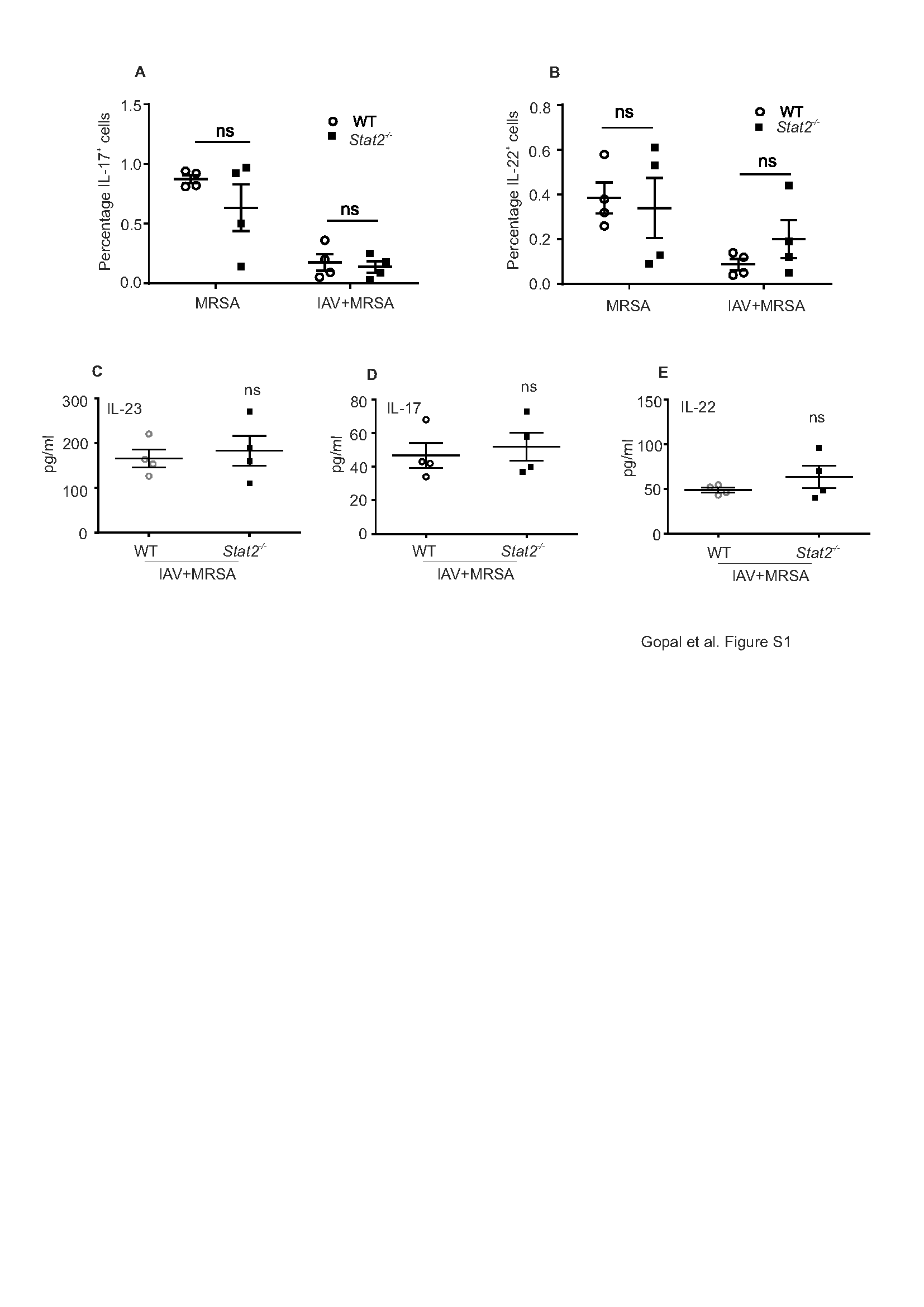

Supplement: FIGURE S1 — No differences in Th17 response between WT and Stat2−/− mice following MRSA, and influenza-MRSA super-infection. WT or Stat2−/− mice were infected with 100 PFU of influenza A/PR/8/H1N1 or PBS by oropharyngeal aspiration for 6 days then challenged with 5 × 10^7^ cfu of MRSA or PBS by oropharyngeal aspiration for one additional day. Frequency of (A) IL-17+ or (B) IL-22+ cells were determined by flow cytometry. The percentage of IL-17+ and IL-22+ cells were calculated from the percentage of CD3+CD4+ T cells. WT or Stat2−/− mice were infected with influenza-MRSA as described above. Lung lobes were homogenized with PBS, and the levels of (C) IL-23, (D) IL-17 were measured by Luminex analysis, and (E) IL-22 levels measured by ELISA. Data are represented as mean ± SEM. Data analyzed using two-tailed Student's t-test, ns, not significant. [file Image_1.TIFF]

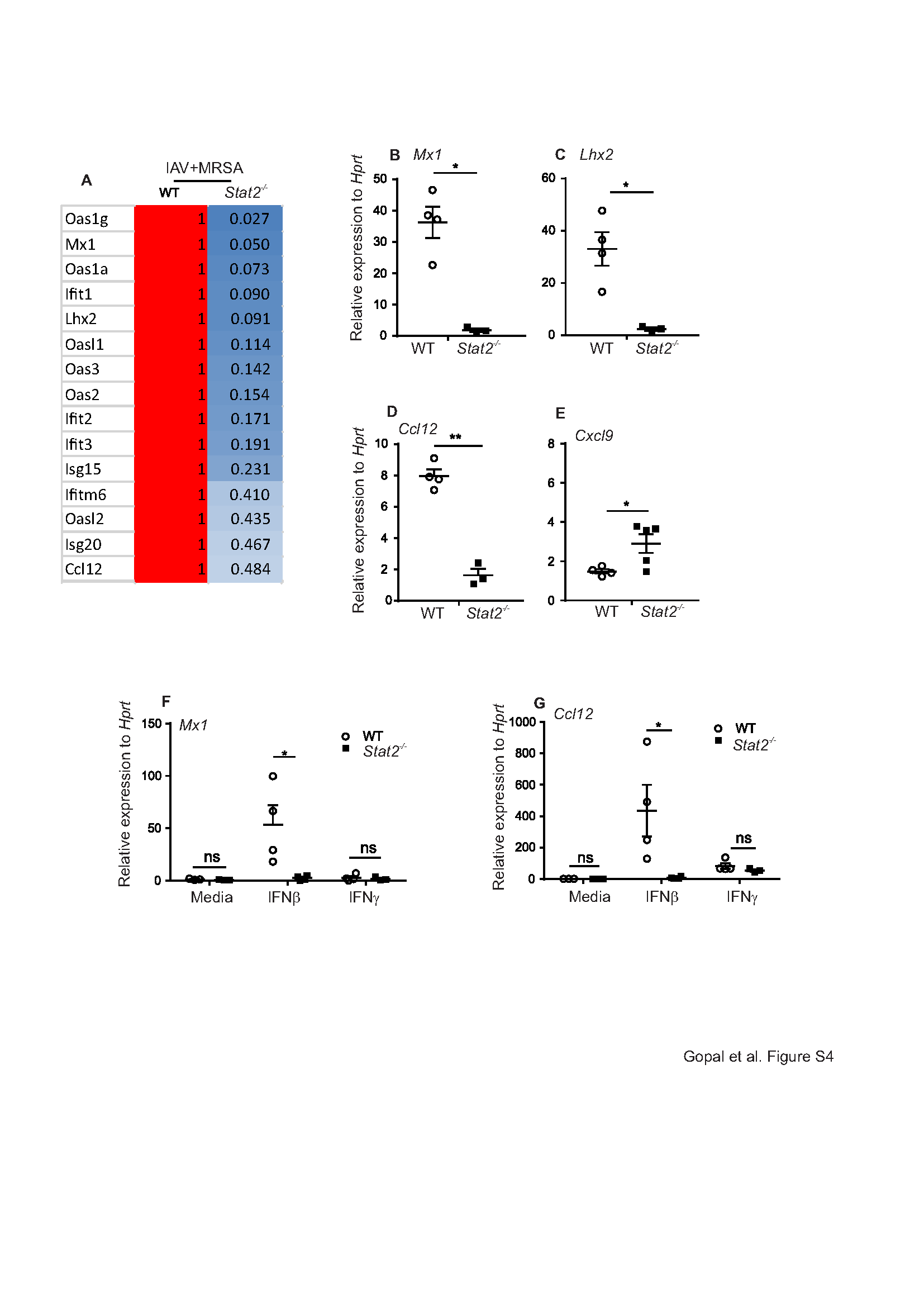

Supplement: FIGURE S4 — Stat2−/− mice have deficient type I IFN responses with preserved type II IFN responses. (A) WT and Stat2−/− male 6–8 weeks mice were infected with 100 PFU of influenza A/PR/8/H1N1 by oropharyngeal aspiration for 6 days then challenged with 5 × 10^7^ cfu of MRSA by oropharyngeal aspiration for one additional day. Overall gene expression was measured in lung by RNAseq analysis, Heat-map representing the fold induction of Interferon stimulated genes (ISGs) from WT and Stat2−/− mice following influenza-MRSA super-infection, N = 4 per group. WT or Stat2−/− mice were infected with either 100 PFU of influenza A PR/8/H1N1 or influenza for 6 days and then challenged with 5 × 10^7^ cfu of MRSA by oropharyngeal aspiration for one additional day, (B) Mx1, (C) Lhx2, and (D) Ccl12, (E) Cxcl9 gene expression relative to Hprt was analyzed by RT-PCR. N = 3–4 per group. Bone marrow-derived dendritic cells (1 × 10^6^) were cultured in vitro and stimulated in vitro with IFNβ (6.5 units/mL) or IFNγ (20 ng/mL) for 24 h, (F) Mx1, (G) Ccl12 gene expression in cell RNA was analyzed by qRT-PCR. The cells were treated in triplicates. Each data point from panel F and G were the number of replicates per treatment. Data are represented as mean ± SEM. Data analyzed using two-tailed Student's t-test, *p ≤ 0.05, **p ≤ 0.01, ns, not significant. [file Image_4.TIFF]

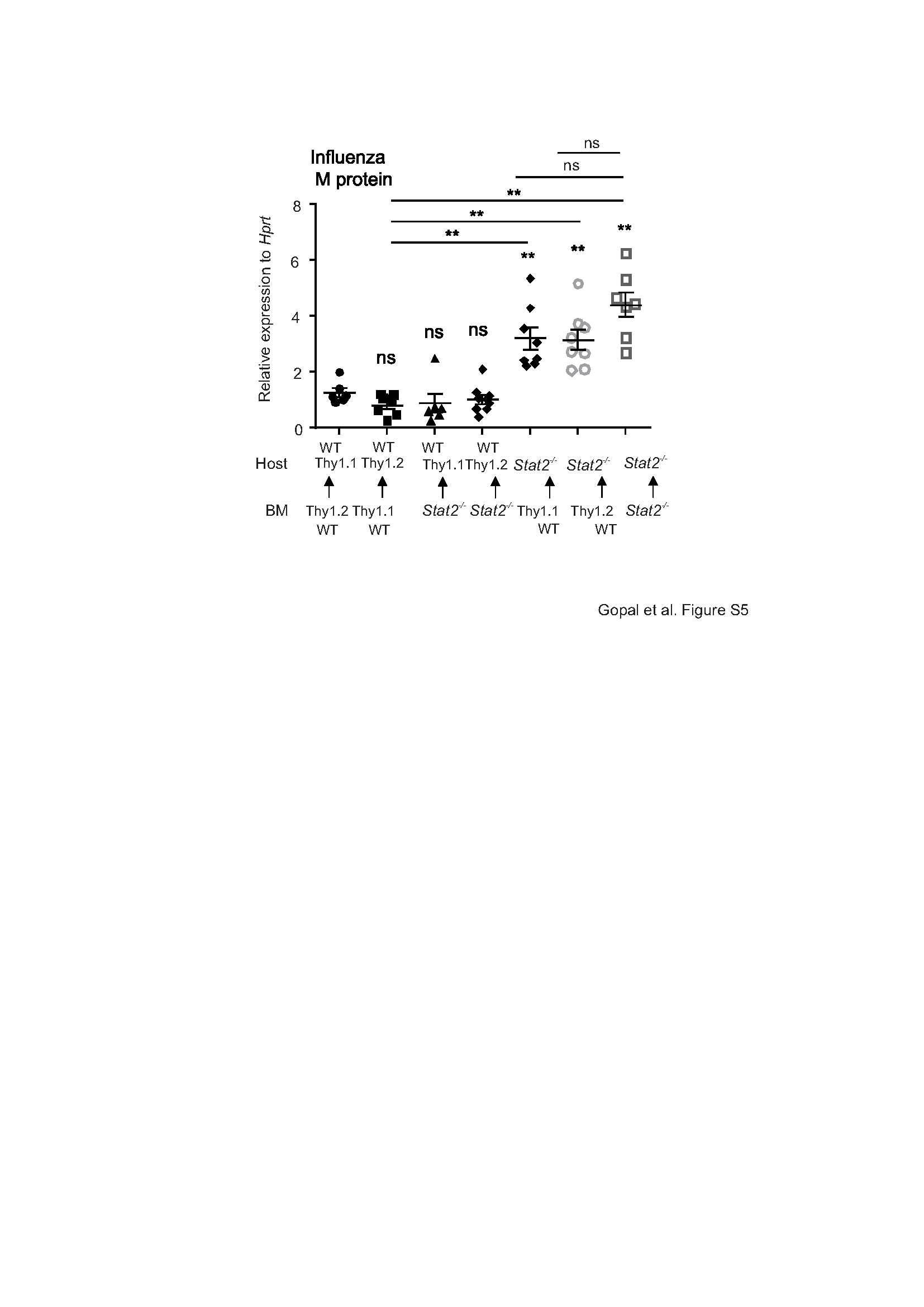

Supplement: FIGURE S5 — Increased influenza burden in cells from non-hematopoietic compartments of Stat2−/− mice. WT BMC (Thy1.1 host, Thy 1.2 BM or Thy 1.2 host, Thy 1.1 BM), Stat2−/− BMC (Stat2−/− host, Stat2−/− BM), Hematopoietic Stat2−/− BMC mice (Thy 1.1 or Thy 1.2 host, Stat2−/− BM), non-hematopoietic Stat2−/− BMC (Stat2−/− host, Thy 1.1 or Thy 1.2 BM) were generated as described in methods. These mice were infected with 100 PFU of influenza for 6 days then challenged with 5 × 10^7^ cfu of MRSA for one additional day. Mice were sacrificed and RNA expression of Influenza M protein was measured from the lungs by PCR, N = 6–9 per group. Data are represented as mean ± SEM. Data analyzed using One way ANOVA followed by Bonferroni test for multiple comparisons, **p ≤ 0.01, ns, not significant. [file Image_5.TIFF]
